# Mechanical Performance of Gravelly Soil Stabilized with Recycled Polypropylene Fiber and Polyurethane

**DOI:** 10.3390/polym18131594

**Published:** 2026-06-26

**Authors:** Pei Zuan, Jiali Feng, Pingcuo Langjia, Xinghong Liu

**Affiliations:** 1 School of Environment and Civil Engineering, Chengdu University of Technology, Chengdu 610059, China; peizuan@cdut.edu.cn; 2 Tibet Geological Second Team Mining Co., Ltd., Lhasa 850000, China; tbf1715746591@163.com; 3 PowerChina Kunming Engineering Corporation Limited, Kunming 650032, China; l1394019007@gmail.com

**Keywords:** polyurethane, recycled polypropylene fiber, stabilized gravel soil, erosion resistance, mountainous rockfall barrier

## Abstract

Gravel soil used as backfill behind rockfall barriers in mountainous roads can extend structural service life and support sustainable resource utilization. However, rainfall-induced erosion may cause soil loss and reduce its buffering capacity. The fibers are short discrete fibers with a length of approximately 12 mm and an average diameter of 32.7 μm, corresponding to an aspect ratio of approximately 367. Reinforcement is achieved through fiber–soil interaction mechanisms, including particle bridging, interfacial friction, and pull-out resistance. The effects of polyurethane and fiber contents on compressive strength, shear strength, and impact resistance were evaluated using response surface methodology. Scanning electron microscopy was used to examine the microstructural features associated with the reinforcement mechanisms, and engineering-scale model tests were conducted to assess erosion and impact resistance under representative service conditions. The results show that polyurethane and fibers produce significant nonlinear enhancement effects on the mechanical properties of gravel soil, mainly through their individual contributions, whereas their interaction is limited. Multi-objective optimization indicates that the optimal mixture contains 6.8% polyurethane and 0.19% fiber, with prediction errors below 5%. The unconfined compressive strength of the gravelly soil increased from 107.6 kPa to 931.5 kPa, representing a 765.7% increase. Cohesion increased from 23.4 kPa to 83.44 kPa, representing a 256.4% increase. The internal friction angle increased from 43.4° to 61.23°, corresponding to a 41.08% increase. Under 1 h of intense rainfall erosion, the stabilized soil exhibited only slight surface particle detachment and maintained overall integrity. In impact tests, the velocity attenuation rate reached 65.6–71.4%. The proposed material provides a sustainable solution for improving buffer layers in rockfall barriers.

## 1. Introduction

Rockfall barriers are widely used in mountainous transportation corridors, including highways, railways, and tunnel entrances, to mitigate hazards caused by falling rocks and rolling debris. These systems are critical for protecting transportation infrastructure and ensuring personnel safety in highly vulnerable mountainous regions [1]. Among their components, the buffer layer plays a central role in impact energy attenuation, and its mechanical performance largely determines the protective efficiency and long-term service life of the entire rockfall protection system [2,3].

In engineering practice, buffer materials are commonly derived from locally available soils or recycled resources, such as gravel soil, sand, used tires, and polymer foams [4,5,6,7]. Tire-derived materials can absorb impact energy effectively, but their long-term performance may deteriorate under ultraviolet aging, environmental exposure, and rainfall-induced degradation [8]. Expanded polystyrene (EPS) and similar polymer foams have also been used as lightweight cushioning materials; however, their poor degradability and recycling difficulties raise environmental concerns [9,10]. Compared with these artificial cushioning materials, gravel soil and sand are more widely available, cost-effective, and easier to construct in mountainous areas. However, these granular materials generally exhibit weak interparticle cohesion, and particle migration or surface erosion may occur under rainfall and wind action, resulting in a progressive loss of buffering capacity [11,12]. Therefore, improving the structural stability, erosion resistance, and impact-buffering performance of granular buffer materials remains a key challenge in rockfall protection engineering [13,14,15].

Fiber reinforcement and polymer stabilization have been widely investigated as effective approaches for improving the mechanical behavior of soils and granular materials. Fiber reinforcement can improve strength and ductility mainly by mobilizing interfacial friction, mechanical interlocking, tensile restraint, and crack-bridging effects [16,17,18,19]. Previous studies have shown that basalt fibers and polypropylene fibers can enhance the shear strength and deformation resistance of soils and sands [20,21], and can also reduce shrinkage-induced cracking in fine-grained soils [22,23]. In addition, recycled fibers have attracted increasing attention because they can improve toughness and ductility while promoting the reuse of waste polymer materials [24]. Polymer stabilization, by contrast, mainly improves soil behavior by strengthening interparticle bonding, filling pores, and enhancing the integrity of granular assemblies. Among polymer stabilizers, polyurethane has shown strong potential for improving cohesion, stiffness, and dynamic performance in gravelly and sandy soils [25,26].

Although previous studies have provided useful insights into fiber-reinforced soils and polymer-stabilized granular materials, several issues remain insufficiently addressed. First, most studies have focused on either fiber reinforcement or polymer stabilization alone, whereas the combined use of recycled fibers and polymer binders in coarse-grained gravel soil has received relatively limited attention. Second, existing studies have mainly emphasized basic mechanical properties, such as compressive strength and shear strength, while the performance of stabilized granular buffer materials under rainfall erosion and rockfall impact loading has not been sufficiently investigated. Third, the use of recycled polypropylene fibers in rockfall barrier buffer layers remains limited, particularly in terms of mixture optimization, microstructural interpretation, erosion resistance, and impact-buffering behavior. These research gaps restrict the development of sustainable and durable granular buffer materials for rockfall protection systems.

Against this background, this study applies a composite stabilization method using waterborne polyurethane and recycled polypropylene fibers to improve gravel soil used as a buffer-layer material behind rockfall barriers. Waterborne polyurethane is a liquid polymer that can be mixed into the granular matrix and cured in situ, forming bonding films and interparticle bonding bridges. Compared with rigid cementitious binders, it can enhance interparticle cohesion while partly maintaining the deformability of the soil skeleton, which is beneficial for impact buffering and erosion resistance. Recycled polypropylene fibers act as randomly distributed short discrete reinforcements and may provide tensile restraint and crack-bridging capability. Thus, polymer bonding and fiber reinforcement are expected to improve particle-scale interaction and macroscopic structural integrity through their respective mechanisms under hydraulic and impact loading.

Recycled polypropylene fibers act as randomly distributed short discrete reinforcements, providing tensile resistance and crack-bridging capability. Polymer bonding and fiber reinforcement contribute to the improvement of particle-scale interaction and macroscopic structural integrity through their respective mechanisms under hydraulic and impact loading. Mechanical behavior under varying material contents is evaluated in terms of compressive strength, shear strength, and impact resistance using response surface methodology. Microstructural mechanisms are analyzed using environmental scanning electron microscopy, and large-scale model tests are conducted to assess erosion and impact performance under representative conditions.

The system is thus developed as a fiber–polymer-reinforced granular medium capable of improving strength, deformation resistance, and energy dissipation for buffer layers in rockfall protection systems.

## 2. Materials and Methods

### 2.1. Materials

#### 2.1.1. Gravel Soil

**Figure 1:**
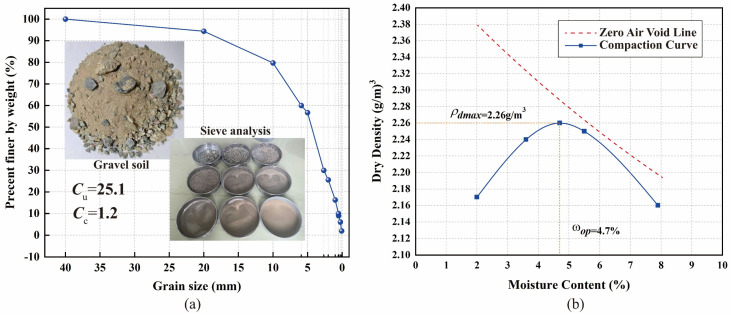
(**a**) Particle-size distribution curve; (**b**) compaction test results.

The gravel soil used in this study was collected from the backfill behind a rockfall barrier in Tibet, China. The particle-size distribution curve obtained from sieve analysis and the compaction test results are shown in Figure 1. The results indicate that the soil contains a high proportion of coarse particles, with gravel particles larger than 4.75 mm accounting for more than 50% of the total mass. Therefore, the material can be classified as a typical coarse-grained soil. The fine-particle content, defined as particles smaller than 0.075 mm, is less than 5%, indicating a low proportion of silt and clay. The maximum dry density and optimum moisture content of the gravel soil are 2.26 g/cm^3^ and 4.7%, respectively.

#### 2.1.2. Water-Based Polyurethane

**Figure 2:**
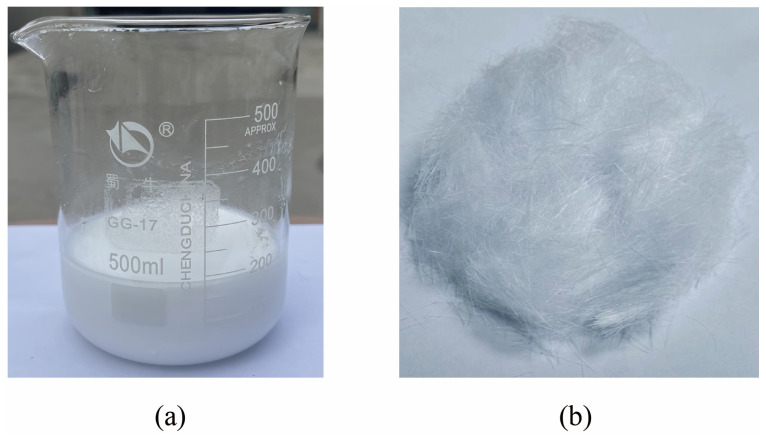
Physical appearance of the recycled polypropylene fibers and waterborne polyurethane: (**a**) waterborne polyurethane; (**b**) recycled polypropylene fibers.

Waterborne polyurethane is a polyurethane material that uses water as the dispersion medium. The waterborne polyurethane used in this study was a colorless transparent liquid with a solid content of 99.9%, a density of 1.0 g/cm^3^, an NCO content of 22.5%, and a viscosity of 2000–6000 mPa·s, as shown in Table 1. The NCO-containing polyurethane prepolymer provides reactive groups for subsequent curing and bonding, while its fluidity allows it to be mixed with gravelly soil and to coat particle surfaces under mechanical stirring. Owing to its adhesion, flexibility, and water stability, this material has been used for soil reinforcement and improvement [27]. Previous studies have reported that waterborne polyurethane can improve soil stability by modifying particle contacts and interparticle bonding [28]. Its slight volumetric expansion during curing may also contribute to pore filling and improved contact conditions between particles [29]. The waterborne polyurethane used in this study is shown in Figure 2a.

#### 2.1.3. Recycled Polypropylene Fiber

It is characterized by low density, relatively high tensile strength, corrosion resistance, and chemical stability [30,31,32]. The recycled polypropylene fibers used in this study have a length of 12 mm and an average diameter of approximately 32.7 μm. The corresponding aspect ratio is approximately 367. The fiber diameter was provided by the supplier and represents a nominal average specification rather than an exactly controlled value for each individual fiber. Based on their geometry, the fibers can be classified as short discrete fibers commonly used in fiber-reinforced soil applications.

Due to their relatively high aspect ratio, the fibers are able to develop effective interaction with the soil matrix, forming a three-dimensional reinforcement network. This reinforcement improves soil behavior through particle interlocking, interfacial friction, and tensile resistance provided by fiber bridging, thereby enhancing the overall strength and deformation resistance of the soil composite. Its relatively rough surface promotes effective interfacial friction with soil particles. When incorporated into soil, recycled polypropylene fibers restrict the relative displacement of soil particles through bridging and pull-out resistance mechanisms [33], thereby improving the tensile strength and shear performance of the soil [34]. In addition, these fibers can delay crack initiation and propagation [35], enhance ductility and energy dissipation capacity, and reduce the brittleness of the material. The recycled polypropylene fibers used in this study are shown in Figure 2b.

Compared with virgin polypropylene fibers, recycled polypropylene fibers have slightly lower tensile strength, but their elastic modulus, density, and elongation at break are generally comparable, as shown in Table 2. Moreover, they can provide comparable reinforcement effects in soil improvement applications [36]. Therefore, using recycled polypropylene fibers can promote the reuse of waste polymer materials, reduce the consumption of virgin resources, and provide environmental and economic benefits.

### 2.2. Methods

**Figure 3:**
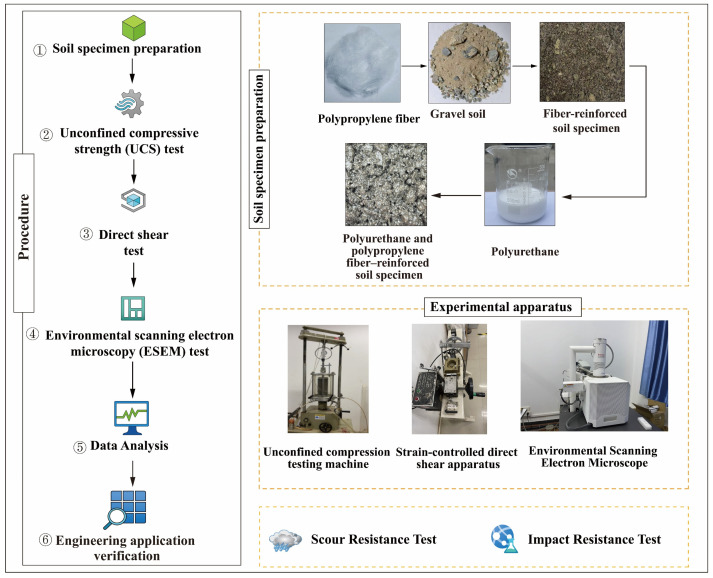
Research framework. The arrows indicate the experimental sequence from step ① to step ⑥. The dashed orange boxes denote the soil specimen preparation and experimental apparatus, respectively. The icons represent different test stages: soil specimen preparation (cube), UCS test (gear), direct shear test (shear symbol), ESEM test (microscope), data analysis (monitor), and engineering application verification (magnifier).

This study adopted a research framework integrating single-factor analysis, microscopic characterization, response surface-based multi-objective optimization, and model-test validation, as shown in Figure 3. In the single-factor tests, recycled polypropylene fiber and polyurethane were independently controlled as experimental variables to obtain baseline data and define the parameter ranges for response surface methodology. Microscopic tests were then conducted to clarify the underlying reinforcement mechanisms of the two materials. Based on the preliminary results, response surface methodology was used to further examine variable interactions, establish fitted models, and perform optimization-based decision making to determine the optimal parameter combination. Finally, model tests were conducted using the optimal mixture proportion for validation and further analysis.

To improve the dispersion uniformity and interfacial compatibility among gravel soil, recycled polypropylene fibers, and waterborne polyurethane, a staged mixing procedure was adopted during specimen preparation. The recycled polypropylene fibers were not directly dispersed in the polyurethane liquid phase. Instead, the fibers were first manually loosened and then gradually added into the dry gravel soil during mechanical mixing, which promoted preliminary fiber dispersion and reduced local fiber entanglement. Subsequently, water was added according to the optimum moisture content and mixed thoroughly. Finally, waterborne polyurethane was introduced into the fiber–soil mixture and continuously mixed, allowing the polymer emulsion to coat soil particles and contact fiber surfaces. During curing, polyurethane gradually formed bonding films and cemented bridges among soil particles and fibers, thereby improving the integrity of the fiber–polymer–soil system. No additional surfactants, dispersants, coupling agents, cellulose fibers, or silica nanoparticles were used in this study. Although cellulose fibers and silica nanoparticles may further improve interfacial compatibility and environmental performance, their effects on the mechanical behavior and durability of recycled polypropylene fiber–polyurethane stabilized gravel soil require further investigation. In addition, slight local fiber entanglement may still occur at relatively high fiber contents, which may partly explain the limited improvement or reduction in mechanical properties when excessive fibers are added.

Fiber distribution quality was evaluated through a combination of visual inspection and post-test observations. During the dry-mixing stage, the mixture was visually examined and any obvious fiber agglomerations were manually dispersed before the addition of water and polyurethane. After curing and mechanical testing, representative failure surfaces were inspected to assess the spatial distribution of fibers within the soil matrix and identify potential local clustering. Environmental scanning electron microscopy (ESEM) was further employed to characterize the microstructural interactions among fibers, polyurethane, and soil particles. The observations indicated that the staged mixing procedure effectively minimized large-scale fiber agglomeration and promoted interactions among the fiber, polymer, and soil phases. It should be noted that the assessment of fiber distribution in this study was primarily qualitative, and no independent quantitative method was used to characterize the three-dimensional spatial distribution of fibers.

The experimental program included unconfined compressive strength tests, direct shear tests, environmental scanning electron microscopy (ESEM) observations, and physical model tests. The experimental procedures followed standardized testing methods where applicable. The unconfined compressive strength tests and direct shear tests were conducted in accordance with ASTM D2166/D2166M [37] and ASTM D3080 [38], respectively. The compaction characteristics were determined following ASTM D698 [39]. The environmental scanning electron microscopy (ESEM) observations were performed under controlled vacuum and imaging conditions to ensure consistent microstructural characterization. Model tests for erosion and impact response were designed based on previous studies on granular buffer materials for rockfall protection systems, with appropriate scaling to simulate representative engineering conditions. All mechanical tests were conducted at least three times, and the average values were reported to ensure reliability and reproducibility of the results.

To determine the approximate range of mixture proportions, the fiber and polymer contents were selected mainly based on previous studies [17,26,40,41], as well as the need to evaluate the dosage-dependent effects of the two additives. As shown in Table 3, four fiber contents and six polyurethane contents were designed to investigate the effects of different additive dosages on material performance. The polyurethane content was set within the range of 0–10% to cover untreated, low-, medium-, and relatively high-dosage conditions, so as to examine whether increasing polyurethane content would continuously improve the soil properties or produce unfavorable effects at excessive dosages. The recycled polypropylene fiber content was set within the range of 0–0.3%, considering both the potential reinforcement effect of fibers and their dispersion behavior in gravelly soil; excessive fiber content may lead to fiber agglomeration and reduced specimen uniformity. The groups without polyurethane or fibers were used as control groups to evaluate the improvement effect of each additive. In this study, “%” denotes the mass ratio of polyurethane or fiber to the dry soil sample. To minimize experimental error, all specimens were prepared at the optimum moisture content and subjected to subsequent mechanical tests under identical curing conditions.

## 3. Analysis of Single-Factor and Microscopic Test Results

### 3.1. Single-Factor Analysis

#### 3.1.1. Effects of Polyurethane and Recycled Polypropylene Fibers on Compressive Strength

**Figure 4:**
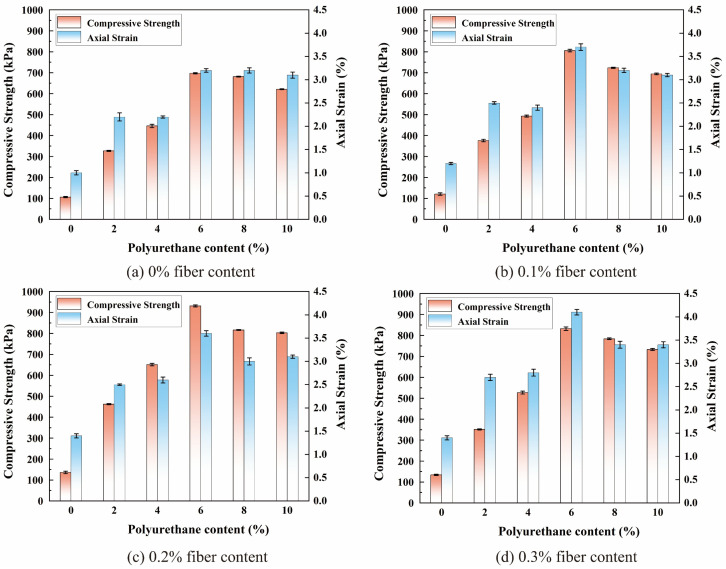
Unconfined compressive strength and axial strain at peak stress of specimens with different polyurethane and recycled polypropylene fiber contents.

The unconfined compressive strength and axial strain at peak stress of the specimens with different polyurethane and recycled polypropylene fiber contents are shown in Figure 4. The results indicate that both polyurethane and recycled polypropylene fibers significantly improve the unconfined compressive strength of the soil.

At a fixed fiber content, the unconfined compressive strength generally increased initially and then decreased with increasing polyurethane content. As the polyurethane content increased from 0% to 6%, the compressive strength of the specimens increased markedly and reached its maximum at 6%. However, when the polyurethane content was further increased to 8% and 10%, the strength decreased to varying degrees. This indicates that an appropriate polyurethane content can improve the integrity and bearing capacity of gravel soil, whereas excessive polyurethane may reduce its compressive performance. Meanwhile, the axial strain generally increased with increasing polyurethane content and reached a relatively high level at approximately 6%, suggesting that polyurethane not only enhances specimen strength but also improves deformation compatibility.

At a fixed polyurethane content, the compressive strength generally increased as the fiber content increased from 0% to 0.2%. However, when the fiber content was further increased to 0.3%, the strength decreased. This reduction may be attributed to fiber agglomeration at excessive fiber contents, where clustered fibers form weak planes within the specimens and consequently reduce strength [42]. Overall, the polyurethane content should be controlled within 6–8%, and the fiber content should be maintained within 0.1–0.2%. Based on the single-factor analysis, the specimens prepared with 6% polyurethane and 0.2% fiber exhibited the highest compressive strength and favorable deformation capacity.

#### 3.1.2. Effects of Polyurethane and Recycled Polypropylene Fibers on Shear Performance

**Figure 5:**
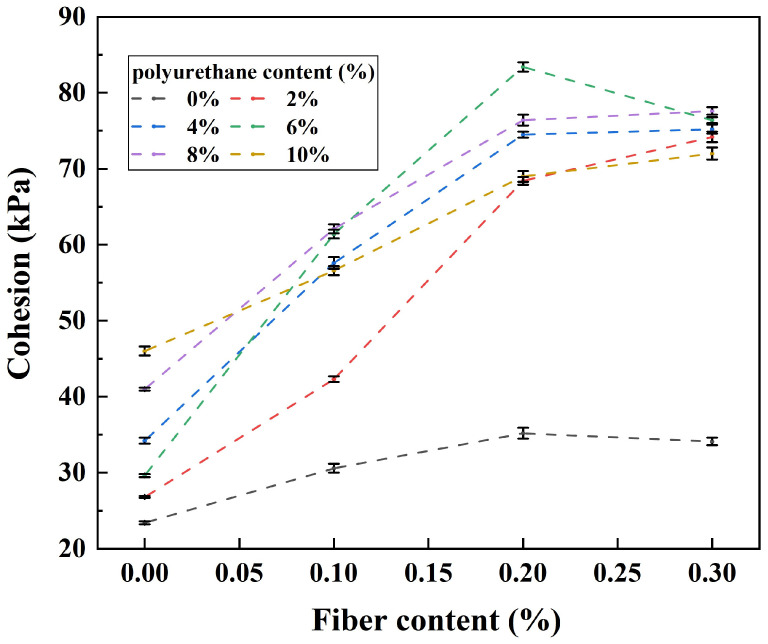
Variation in cohesion of specimens with different polyurethane and recycled polypropylene fiber contents.

Cohesion and internal friction angle are key parameters for characterizing the shear strength of soils. As shown in Figure 5, at a given fiber content, cohesion increased markedly with increasing polyurethane content. The most pronounced increase occurred as the polyurethane content increased from 0% to 6%. When the polyurethane content was further increased to 8% and 10%, the increase in cohesion became less pronounced, and slight decreases were observed in some cases, indicating a saturation effect in the cementation provided by polyurethane. At a given polyurethane content, cohesion generally increased with increasing fiber content and remained relatively high at fiber contents of 0.2–0.3%. The specimen containing 6% polyurethane and 0.2% fiber exhibited the highest cohesion, indicating that polyurethane cementation and fiber reinforcement jointly improved the cohesion through their respective mechanisms at this mixture proportion.

**Figure 6:**
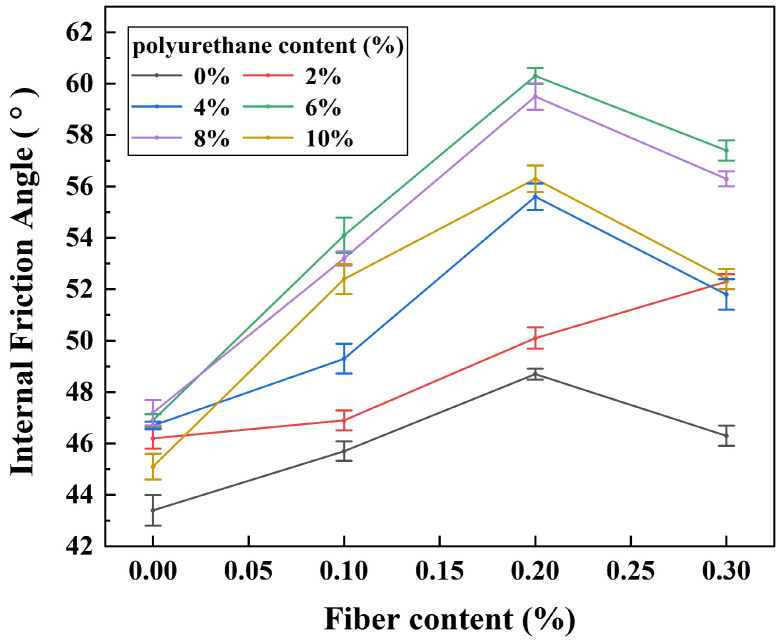
Variation in the internal friction angle of specimens with different polyurethane and recycled polypropylene fiber contents.

As shown in Figure 6, the internal friction angle generally increased initially and then decreased with increasing polyurethane and fiber contents. The relatively large internal friction angle may be related to the coarse-grained characteristics of the gravelly soil used in this study. Compared with fine-grained soils, gravelly soil generally contains larger particles with rougher surfaces and more pronounced particle contacts, which may increase interparticle friction, mechanical interlocking, and dilatancy during shearing. These characteristics can lead to a steeper Mohr–Coulomb failure envelope and thus a relatively large internal friction angle. When the fiber content increased from 0% to 0.2%, the internal friction angle increased markedly, suggesting that an appropriate fiber content may enhance the apparent frictional resistance and structural stability of the soil matrix. However, when the fiber content was further increased to 0.3%, the internal friction angle decreased, which may be attributed to fiber agglomeration, restricted particle contact, and the formation of local weak zones. Similarly, the internal friction angle reached its maximum at a polyurethane content of 6% and then decreased with further increases in polyurethane content. This decrease may be related to excessive polymer coating, which could weaken direct particle contact and reduce the apparent frictional contribution. Overall, the polyurethane content should be controlled within 6–8%, and the fiber content should be maintained at approximately 0.2%.

### 3.2. Environmental Scanning Electron Microscopy (ESEM) Test

This section employs environmental scanning electron microscopy (ESEM) to investigate the reinforcement mechanism of gravel soil treated with polyurethane and recycled polypropylene fibers.

#### 3.2.1. Reinforcement Mechanism of Polyurethane

**Figure 7:**
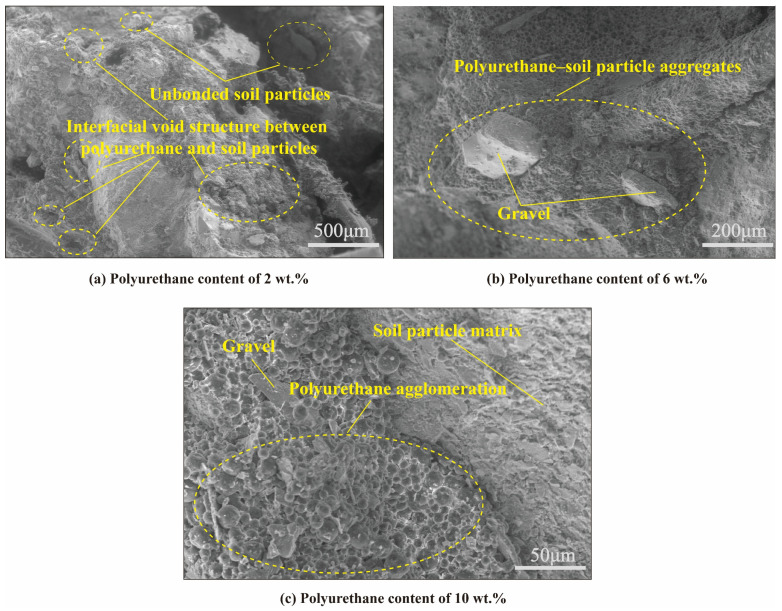
Environmental scanning electron microscopy (ESEM) images of cured gravelly soil stabilized with different polyurethane contents at a fixed fiber content of 0.2 wt.%: (**a**) 2 wt.% polyurethane; (**b**) 6 wt.% polyurethane; (**c**) 10 wt.% polyurethane.

Based on the single-factor analysis, the optimal fiber content was determined to be 0.2%. At this fiber content, gravel soil specimens with polyurethane contents of 2%, 6%, and 10% were selected for environmental scanning electron microscopy (ESEM) observation. The corresponding microstructural characteristics are shown in Figure 7.

At low polyurethane contents, numerous pores remained between gravel soil particles, and some free particles were present. At this stage, polyurethane mainly existed as surface coatings and local bonds. It did not effectively fill the interparticle pores or form a continuous structure, and potential weak planes remained at particle contact interfaces, resulting in a limited reinforcement effect. At a polyurethane content of 6%, the polymer gradually filled the interparticle pores and formed polymer aggregates. The number of pores decreased markedly, and free particles almost disappeared. Polyurethane improved particle contact through coating, bonding, and filling effects, while also enhancing the interfacial interaction between soil particles and fibers. When the polyurethane content increased to 10% (Figure 7c), polyurethane tended to become saturated within the observed field. In addition to filling pores, excess polymer accumulated on particle surfaces and formed relatively thick coating layers. Combined with the observed reduction in mechanical performance, this phenomenon suggests that excessive polyurethane may alter particle contact conditions and reduce the effectiveness of the reinforcement mechanism. However, this interpretation is based on qualitative ESEM observations and should be regarded as a possible explanation rather than a definitive mechanism. Further quantitative investigations are required to verify the underlying cause of strength reduction at high polyurethane contents.

**Figure 8:**
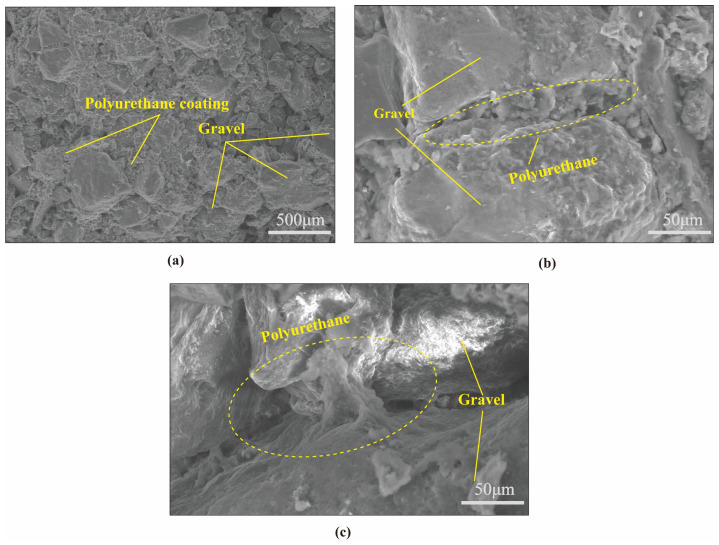
Polyurethane Modification Mechanisms for Gravel Soil; (**a**) Surface Coating; (**b**) Pore Filling; (**c**) Particle Bonding.

In summary, polyurethane reinforces gravel soil mainly through coating, bonding, and pore filling, as shown in Figure 8. Polyurethane gel coats particle surfaces and fills pores. Under external loading, it forms bridging connections that transform initially loose particle contacts into cemented structures, thereby improving the internal soil structure and enhancing overall stability.

#### 3.2.2. Reinforcement Mechanism of Recycled Polypropylene Fibers

**Figure 9:**
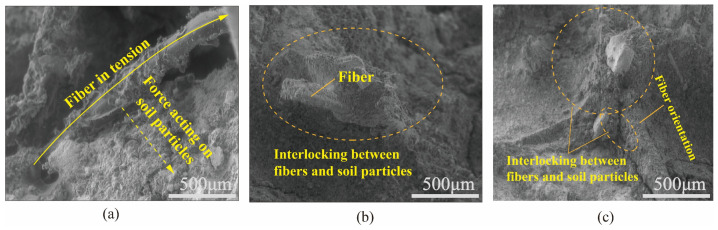
Frictional and Interlocking Interactions Between Fibers and Soil Particles: (**a**) Frictional Interaction; (**b**) Fiber Embedding and Interlocking Interaction; (**c**) Interlocking Interaction.

Based on the preceding analysis, gravelly soil specimens with the preferred mixture proportion of 0.2% fiber and 6% polyurethane were examined using environmental scanning electron microscopy (ESEM). Based on the ESEM observations, the possible reinforcement behavior of fiber-reinforced gravelly soil may be associated with frictional interaction and interlocking between fibers and soil–particle aggregates, fiber bridging, and stress transfer. In general, the shear strength of granular soil is closely related to interparticle friction, mechanical interlocking, and interfacial interactions [43]. In the stabilized soil, fibers may contribute to the shear resistance of the fiber–soil interface through friction and anchorage with soil particles and polyurethane-induced particle aggregates. When the stabilized soil is subjected to external loading, fiber pull-out may generate shear resistance along the fiber surfaces and exert reaction forces on surrounding particles. This process may enhance interparticle locking and contribute to strength improvement, as shown in Figure 9a. The presence of polyurethane may further improve the interfacial contact between fibers and soil particles.

In addition, particle aggregates are often attached to the fiber surfaces, altering the originally smooth surface morphology of the fibers. When the aggregates are small, they increase friction between the fibers and surrounding particles. When the aggregates are large, they interlock with the surrounding particles during sliding under loading, requiring the particles to either lift over the aggregates or undergo shear failure before further sliding can occur. This further improves the shear strength of the soil, as shown in Figure 9b,c.

At relatively high fiber contents, a fiber network can form within the soil. Under external loading, fibers surrounding the particles exert an inward confining force, similar to a “hoop effect,” thereby improving the overall stability of the soil and enhancing cohesion.

**Figure 10:**
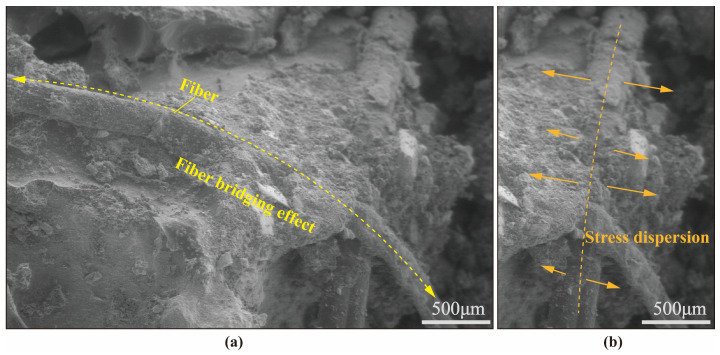
Bridging and Stress Dispersion Effects: (**a**) Bridging Effect (yellow dashed lines and arrows indicate the fiber and bridging direction); (**b**) Stress Dispersion Effect (yellow arrows indicate the stress dispersion direction).

In addition, fibers provide bridging and stress redistribution effects. When cracks develop in the soil, fibers spanning the two sides of a crack can transfer tensile forces and restrict crack propagation, as shown in Figure 10a, thereby improving soil ductility. This also explains why the stabilized soil can maintain relatively high strength at large strains in the unconfined compressive strength test. Meanwhile, when stress concentration occurs within the soil, fibers can redistribute local stress to the surrounding regions through interfacial interactions, as shown in Figure 10b. This reduces stress concentration in weak zones and improves the overall stress state.

### 3.3. Response Surface Modeling and Multi-Objective Optimization

The preceding single-factor analysis showed that improvements in compressive and shear strengths require the polyurethane content to be controlled within an appropriate range of approximately 6–8%, together with a suitable fiber content of approximately 0.1–0.2%. Although excessive additive contents may improve the local structure to some extent, they can weaken the overall material performance owing to embrittlement or fiber agglomeration.

Based on the parameter ranges determined from the single-factor analysis, this section further examines the individual effects, quadratic effects, and interaction terms of polyurethane and fiber contents on different mechanical indices using response surface methodology. Multi-objective optimization is then performed to determine the optimal mixture proportion that balances strength and deformation performance.

Considering that the response surface design in this study included only 13 experimental runs, including five replicated center-point tests, the experimental dataset was relatively limited. Therefore, a model uncertainty analysis was further supplemented to evaluate the reliability of the fitted models and the robustness of the optimization results. In the replicated center-point tests, the unconfined compressive strength (UCS) fluctuated within the range of approximately 901.2–975.0 kPa, indicating a certain degree of inherent variability in the modified gravelly soil specimens. This variability may be associated with the heterogeneity of the gravelly soil, differences in specimen preparation, fiber dispersion, polyurethane distribution, and variations in the curing process. Therefore, the response surface models in this study were mainly used to identify response trends and high-performance regions within the experimental design space and to provide a statistically meaningful reference for mixture optimization, rather than serving as fully deterministic predictive models.

#### 3.3.1. Response Surface Model Construction and Significance Testing

The response surface experiment was designed using a central composite design (CCD). The number of experimental runs was determined using the calculation formula for response surface design, as shown in Equation (1). In this study, 13 experimental runs were conducted. Polyurethane content (*x*_1_, wt.%) and recycled polypropylene fiber content (*x*_2_, wt.%) were selected as the experimental factors, while unconfined compressive strength (UCS), cohesion, and internal friction angle were used as the response variables. The experimental design and corresponding results are presented in Table 4.

(1) $$N=2^{k}+2k+M_{0}$$

where N denotes the total number of experimental runs in the central composite design, *k* denotes the number of independent variables, with *k* = 2 in this study; *M*_0_ denotes the number of center-point runs, with *M*_0_ = 5 used in this study.

To quantify the nonlinear effects of polyurethane and recycled polypropylene fiber contents on the mechanical properties of improved gravel soil and to determine a reasonable mixture proportion that balances multiple mechanical indices, a quadratic response surface model was established based on the two-factor, five-level experimental data, as expressed in Equation (2):

(2) $$Y=\beta_{0}+\beta_{1}x_{1}+\beta_{2}x_{2}+\beta_{11}x_{1}^{2}+\beta_{22}x_{2}^{2}+\beta_{12}x_{1}x_{2}$$

where Y denotes the response variable, corresponding to unconfined compressive strength, cohesion, or internal friction angle; *x*_1_ and *x*_2_ denote the fiber content and polyurethane content, respectively; and $\beta_{0}$, $\beta_{1}$, $\beta_{2}$, $\beta_{11}$, $\beta_{22}$, and $\beta_{12}$ are the regression coefficients.

In the significance evaluation of the regression models, a larger F-value indicates greater explanatory power for the experimental data. The *p*-value was used to assess model significance. A model was considered statistically significant at *p* < 0.05, whereas *p* < 0.0001 indicated extremely high significance. Insignificant terms (*p* > 0.05) were removed from each model to simplify the model expression. The coefficient of determination (*R*^2^) and adjusted coefficient of determination (Adjusted *R*^2^), calculated after removing insignificant terms, were used to evaluate the fitting accuracy of the model. Values closer to 1 indicate better agreement between the fitted model and the experimental data.

Because quadratic regression models were developed simultaneously for three response variables, namely UCS, cohesion, and internal friction angle, the applicability of the models was comprehensively evaluated to avoid overinterpreting the optimization results due to the limited number of experimental runs. The evaluation was based on analysis of variance, R^2^, adjusted R^2^, comparisons between measured and predicted values, and residual distributions. Among the three models, the UCS and internal friction angle models exhibited relatively high fitting accuracy, with R^2^ values of 0.977 and 0.973 and adjusted R^2^ values of 0.960 and 0.953, respectively. The cohesion model had R^2^ and adjusted R^2^ values of 0.894 and 0.818, respectively. Although these values were lower than those of the UCS and internal friction angle models, the cohesion model was still able to capture the overall variation trend of cohesion with changes in polyurethane and fiber contents.

In addition, the repeated center-point tests provided a basis for estimating model pure error and experimental variability. The fluctuation in UCS values at the center points suggests that the modified gravelly soil was strongly influenced by particle gradation, fiber dispersion, and the curing uniformity of polyurethane. Therefore, in the subsequent optimization analysis, the model-predicted optimal mixture proportion was not interpreted as a unique or absolute optimum. Instead, it was regarded as a statistically supported recommended optimization region within the current experimental range.

#### 3.3.2. Response Surface Analysis of Unconfined Compressive Strength

**Figure 11:**
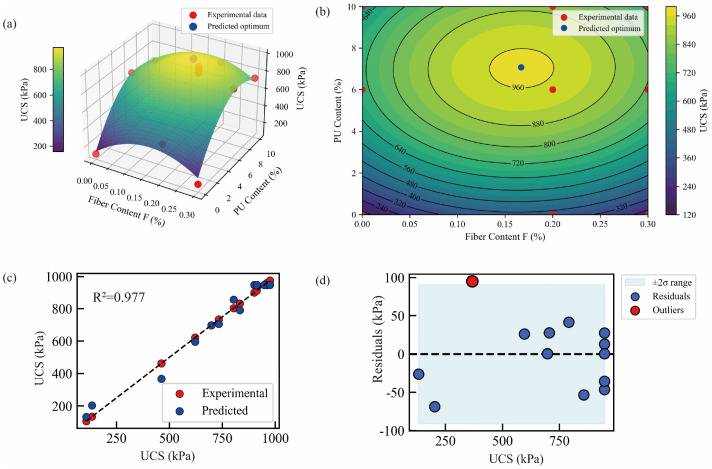
Model visualization for unconfined compressive strength: (**a**) three-dimensional response surface; (**b**) contour plot; (**c**) scatter plot of experimental and predicted values (the dotted line represents the 1:1 ideal fitting line); (**d**) residual plot. UCS denotes unconfined compressive strength (the dotted line represents the zero residual line).

Table 5 presents the coefficient significance and analysis of variance for the unconfined compressive strength regression model, and the corresponding response surface plots are shown in Figure 11. The results indicate that the overall model for unconfined compressive strength is extremely significant, with an F-value of 58.21 and a *p*-value less than 0.0001. This suggests that the quadratic regression model can effectively characterize the effects of polyurethane and fiber contents on the compressive performance of improved gravel soil. The R^2^ and Adjusted R^2^ values are 0.98 and 0.96, respectively, indicating good agreement between the predicted and experimental results and confirming the satisfactory fitting accuracy of the model. The experimental values are generally distributed close to the predicted fitting line (Figure 11c), and the residuals show no obvious systematic deviation (Figure 11d), further demonstrating the predictive reliability of the model.

Regarding the significance of individual terms, the linear terms of fiber content (*x*_1_) and polyurethane content (*x*_2_) were both significant. The F-value for polyurethane content was 100.31, higher than that for fiber content (27.82), indicating that polyurethane had a more pronounced effect on unconfined compressive strength. The quadratic terms *x*_1_^2^ and *x*_2_^2^ were also significant, suggesting that unconfined compressive strength varied nonlinearly with additive content. In contrast, the interaction term was not significant, with a *p*-value of 0.742. This indicates that, within the experimental range considered in this study, the improvement in unconfined compressive strength was mainly governed by the individual main effects and quadratic effects of polyurethane and fiber.

Figure 11a,b shows an arch-shaped response surface and a closed high-value region for unconfined compressive strength. This pattern indicates the presence of an optimum dosage range for both polyurethane and fiber contents, polymer cementation and pore filling are insufficient, and the gravel soil particles remain mainly in loose contact; therefore, the improvement in compressive strength is limited. As the polyurethane content increases, polyurethane gradually forms cemented bridging structures between particles, enhancing interparticle bonding and the overall integrity of the soil, thereby markedly improving the unconfined compressive strength. However, with further increases in polyurethane content, excessive polymer may form a thick coating layer on particle surfaces, weakening direct particle interlocking and frictional contact. As a result, the strength increase tends to slow down or even decline.

The effect of fibers on unconfined compressive strength also shows a nonlinear pattern. An appropriate fiber content can restrict the relative displacement of particles through bridging, tensile restraint, pull-out resistance, and stress redistribution, thereby improving the bearing capacity and deformation compatibility of the material. However, when the fiber content increases beyond the most effective range, the probability of fiber–fiber contact and local overlapping may increase. This can reduce the dispersion uniformity of fibers and decrease the effective bridging efficiency, thereby weakening the marginal reinforcement effect of additional fibers.

#### 3.3.3. Response Surface Analysis of Cohesion

**Figure 12:**
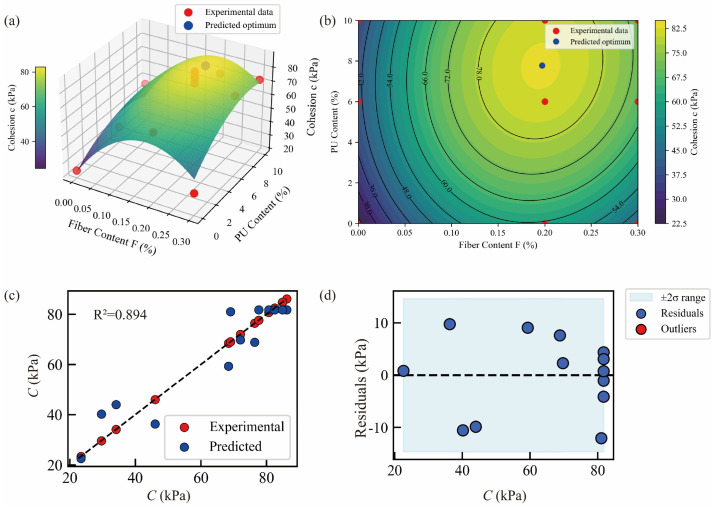
Model visualization for cohesion: (**a**) three-dimensional response surface; (**b**) contour plot; (**c**) scatter plot of experimental versus predicted values (the dotted line represents the 1:1 ideal fitting line); (**d**) residual plot (the dotted line represents the zero residual line).

Table 6 presents the coefficient significance and analysis of variance for the cohesion regression model, and the corresponding response surface plots are shown in Figure 12. The results indicate that the overall cohesion model is statistically significant, with an F-value of 11.77 and a *p*-value of 0.0027. This suggests that the quadratic regression model can describe the effects of polyurethane and fiber contents on cohesion. The R^2^ and Adjusted R^2^ values are 0.90 and 0.82, respectively, indicating reasonable fitting accuracy. The experimental and predicted values show good overall agreement (Figure 12c), and the residuals are relatively concentrated (Figure 12d), indicating that the model can effectively explain the variation in cohesion.

Regarding the model terms, both the linear term of fiber content (*x*_1_) and its quadratic term (*x*_1_^2^) were significant, indicating that fiber content has a pronounced nonlinear effect on cohesion. In contrast, the linear term (*x*_2_) and quadratic term (*x*_2_^2^) of polyurethane content did not reach the 0.05 significance level. However, the response surface trend indicates that polyurethane still contributes to the improvement in cohesion to some extent. The interaction term *x*_1_*x*_2_ was not significant, suggesting that the increase in cohesion mainly results from the independent but cumulative contributions of fiber reinforcement and polyurethane cementation, rather than from a statistically significant interaction effect.

Figure 12 shows that cohesion increased over the low-to-moderate additive range and then tended to stabilize or decrease. Polyurethane enhances interparticle bonding mainly through particle coating, pore filling, and cementation at contact points, thereby improving the overall integrity of the soil. Fibers enhance particle interlocking through interfacial friction, mechanical interlocking, and pull-out restraint.

However, when the polyurethane or fiber content exceeds the appropriate range, the increase in cohesion gradually diminishes. On the one hand, excessive polyurethane may overcoat particle surfaces, weakening direct interlocking between coarse particles. On the other hand, excessive fiber may lead to uneven dispersion or local agglomeration, resulting in stress concentration or the formation of weak structural zones.

#### 3.3.4. Response Surface Analysis of the Internal Friction Angle

**Figure 13:**
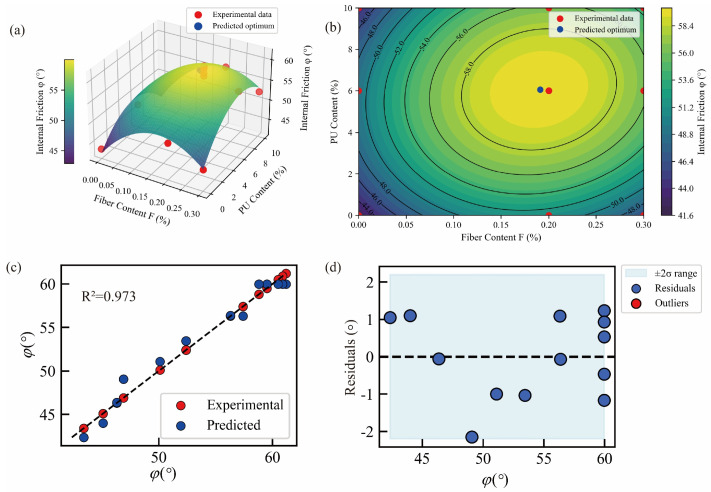
Model visualization for the internal friction angle: (**a**) three-dimensional response surface; (**b**) contour plot; (**c**) scatter plot of experimental versus predicted values (the dotted line represents the 1:1 ideal fitting line); (**d**) residual plot (the dotted line represents the zero residual line).

The statistical results for the internal friction angle model are summarized in Table 7, and the fitted response surface is presented in Figure 13. The model was highly significant, with an F-value of 49.99 and a *p*-value less than 0.0001. This confirms the suitability of the quadratic model for describing the response of the internal friction angle. The *R*^2^ and Adjusted *R*^2^ values are 0.97 and 0.95, respectively, indicating high fitting accuracy. The predicted values agree well with the experimental values (Figure 13c), and the residuals show no obvious systematic deviation (Figure 13d), further confirming the reliability of the response surface model for the internal friction angle.

Regarding the significance results, the linear terms of fiber content (*x*_1_) and polyurethane content (*x*_2_) were both extremely significant, with F-values of 55.98 and 40.53, respectively. This indicates that both modifiers significantly affect the particle contact state and the mobilization of shear resistance. The quadratic terms of both factors were also extremely significant, suggesting that the internal friction angle varies nonlinearly with additive content and exhibits a clear threshold effect. The interaction term *x*_1_*x*_2_ was not significant, indicating that the internal friction angle is mainly governed by the individual main effects and quadratic effects of polyurethane and fiber.

As shown in Figure 13, the response surface of the internal friction angle exhibits a nonlinear trend, characterized by an initial increase followed by a decrease or stabilization. An appropriate polyurethane content can fill some pores, improve particle contact conditions, and promote the formation of particle aggregates, thereby enhancing the stability of the particle skeleton during shearing. Similarly, an appropriate fiber content can restrict particle sliding and rearrangement through friction, entanglement, and mechanical interlocking, allowing shear resistance to be more fully mobilized.

When the polyurethane content is excessive, interparticle contact may gradually shift from hard contact with strong interlocking to polymer-separated contact with weaker friction, thereby reducing frictional resistance. When the fiber content is excessive, fiber entanglement and agglomeration can reduce the internal structural uniformity of the specimen and weaken effective interparticle contact, leading to a decrease in the internal friction angle.

#### 3.3.5. Pareto Non-Dominated Solution Set and Composite Desirability Function-Based Optimization

The preceding sections revealed the variation patterns of unconfined compressive strength, cohesion, and internal friction angle with changes in polyurethane and fiber contents. The results indicate that the optimal regions for different mechanical indices are not fully consistent. If a single index is used as the only optimization objective, trade-offs in other performance indicators may occur, making it difficult to simultaneously satisfy the requirements for compressive bearing capacity, shear stability, and impact resistance. Therefore, the Pareto non-dominated solution set and composite desirability function were further introduced to conduct multi-objective optimization of the material mixture proportions within the Fiber–PU design space.

During the optimization process, unconfined compressive strength, cohesion, and internal friction angle were all set as maximization objectives. Meanwhile, considering material economy, construction feasibility, and the potential structural nonuniformity caused by excessive additive contents, polyurethane and fiber contents were incorporated into the composite evaluation as material-dosage constraints. For unconfined compressive strength, cohesion, and internal friction angle, a “larger-the-better” desirability function was used for normalization, as expressed in Equation (3).

(3) $$d_{i}=\left\{\begin{array}{cc} 0, & Y_{i}\leq Y_{i,\min}\\ {\left(\frac{Y_{i}-Y_{i,\min}}{Y_{i,\max}-Y_{i,\min}}\right)}^{w_{i}}, & Y_{i,\min}<Y_{i}<Y_{i,\max}\\ 1, & Y_{i}\geq Y_{i,\max} \end{array}\right.$$

where $d_{i}$ denotes the individual desirability of the ith mechanical response; $Y_{i}$ is the model-predicted response value; $Y_{i,min}$ and $Y_{i,max}$ are the lower and upper bounds of the corresponding response, respectively; and $w_{i}$ is the weight or shape coefficient of that response.

For polyurethane and fiber contents, a “smaller-the-better” desirability function was introduced as a dosage constraint, as expressed in Equation (4).

(4) $$d_{j}=\left\{\begin{array}{cc} 1, & X_{j}\leq X_{j,\min}\\ {\left(\frac{X_{j,\max}-X_{j}}{X_{j,\max}-X_{j,\min}}\right)}^{w_{j}}, & X_{j,\min}<X_{j}<X_{j,\max}\\ 0, & X_{j}\geq X_{j,\max} \end{array}\right.$$

where $d_{j}$ denotes the individual desirability of the jth material-dosage factor; $X_{i}$ is the material content; $X_{i,min}$ and $X_{i,max}$ are the lower and upper bounds of the corresponding factor, respectively; and $w_{j}$ is the shape coefficient.

The composite desirability was calculated using a weighted geometric mean, as expressed in Equation (5).

(5) $$D=e^{\left(\frac{\sum_{i=1}^{n}\lambda_{i}\ln d_{i}}{\sum_{i=1}^{n}\lambda_{i}}\right)}$$

where *D* denotes the composite desirability, $d_{i}$ denotes the individual desirability, and $\lambda_{i}$ is the weighting coefficient. The value of *D* ranges from 0 to 1, with values closer to 1 indicating better overall coordination of the mixture proportion in multi-objective optimization. Based on the established quadratic response surface models, grid-based prediction was performed within the design space defined by a fiber content of 0–0.3% and a polyurethane content of 0–10%. A composite desirability evaluation was then conducted for cohesion, internal friction angle, and unconfined compressive strength.

The Pareto non-dominated solution set was used to identify effective trade-off solutions in the multi-objective optimization. If one mixture proportion is no worse than another across all objectives and performs better in at least one objective, it is considered to dominate the other mixture proportion. All mixture proportions that are not dominated by any other solution constitute the Pareto non-dominated solution set.

**Figure 14:**
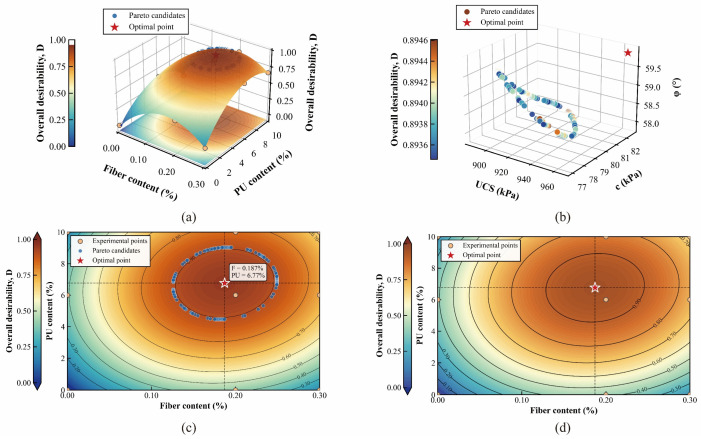
Pareto non-dominated solution set and composite desirability function: (**a**) 3D response surface of overall desirability with respect to fiber content and PU content; (**b**) 3D Pareto optimal front of overall desirability with respect to UCS and cohesion; (**c**) 2D contour plot of overall desirability with experimental points and optimal solution; (**d**) 2D contour plot of overall desirability for verification of the optimal mixture proportion.

Figure 14 shows the distribution of the Pareto candidate solutions, composite desirability function, and optimal solution in the Fiber–polyurethane design space and objective space. The three-dimensional composite desirability response surface and two-dimensional contour plot indicate that a distinct high-value region of composite desirability forms at moderate polyurethane contents and appropriate fiber contents. This suggests that the overall mechanical performance of the improved gravel soil does not continuously increase with increasing polyurethane or fiber content; instead, an optimal balance is achieved within a certain dosage range. The high-desirability region is mainly concentrated at approximately 6–7% polyurethane and 0.18–0.20% fiber, which is generally consistent with the high-strength regions identified from the preceding single-factor tests and response surface analysis.

The composite desirability function optimization indicates that the theoretical optimal mixture proportion is 0.187% fiber and 6.77% polyurethane. At this mixture proportion, the model-predicted cohesion, internal friction angle, and unconfined compressive strength are 82.57 kPa, 59.87°, and 966.22 kPa, respectively, with a composite desirability value (*D*) of 0.95. These results suggest that the modified gravel soil can simultaneously maintain high compressive strength, favorable shear strength parameters, and strong overall performance under the optimal mixture proportion, indicating that the material mixture achieves a favorable balance among multiple optimization objectives.

Considering the material proportioning accuracy and construction feasibility in practical engineering, and the high measured mechanical performance obtained at a fiber content of 0.187% and a polyurethane content of 6.76%, this study selected 0.19% fiber and 6.8% polyurethane as the representative optimal mixture proportion for the subsequent erosion-resistance and impact-buffering model tests.

To verify the reliability of the optimal mixture proportion, soil specimens were prepared using this proportion and subsequently tested. The measured unconfined compressive strength, cohesion, and internal friction angle were 931.5 kPa, 83.44 kPa, and 61.23°, respectively. The errors between the experimental and predicted values were within 5%, confirming the reliability of the predicted mixture proportion. It should be noted that, due to the limited number of response surface experimental runs and the variability observed at the center points, the optimized mixture proportion of 0.19% fiber and 6.8% polyurethane obtained in this study should not be interpreted as a unique or absolute optimum. Rather, it should be regarded as a recommended optimized proportion within the design space considered in this study. This proportion falls within the high-value region of the overall desirability function. In addition, the validation tests showed that the differences between the measured and predicted values of UCS, cohesion, and internal friction angle were all less than 5%, indicating that the optimization result has good robustness and engineering reference value within the investigated factor ranges. Future studies may further improve the confidence and generalizability of the model predictions by increasing the number of experimental runs, expanding the number of replicate tests, and introducing confidence intervals or cross-validation methods.

## 4. Evaluation of Erosion and Buffering Performance

After the optimal mixture proportion was determined in the preceding section, model tests were conducted to evaluate the working performance of gravel soil reinforced with polypropylene fibers and polyurethane. The evaluation focused on erosion resistance and energy absorption capacity.

### 4.1. Overview of the Study Area and the Stone Barrier Prototype

**Figure 15:**
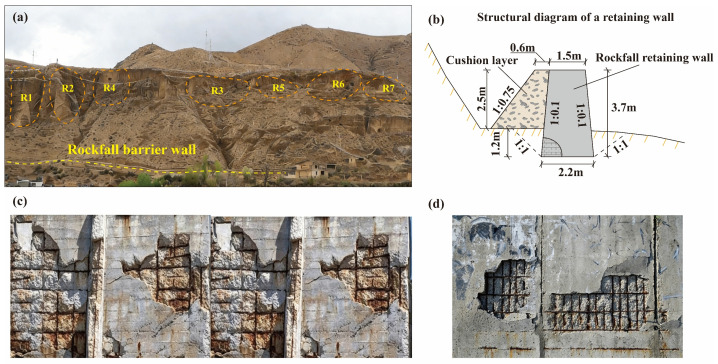
Overview of the study area and rockfall barrier structure: (**a**) overview of the study area (R1–R7 indicate the locations of landslide areas); (**b**) rockfall barrier structure; (**c**) damaged area 1 of the rockfall barrier after rockfall impact; (**d**) damaged area 2 of the rockfall barrier after rockfall impact.

The experiment is based on a prototype slope and rockfall barrier located in Shannan, Tibet. The slope extends approximately 442 m, oriented between 120° and 210°, with a steep upper section (75–90°) and a gentler lower section (25–45°). The maximum height of the slope is 94 m. The slope has been subjected to weathering, erosion, rainfall, and seismic activity, resulting in significant landslides. Field investigations revealed the presence of seven distinct landslides. The topography of the site and the layout of the rockfall barrier are depicted in Figure 15.

The rockfall barrier has a height of 3.7 m, a top width of 1.5 m, and a foundation embedment depth of 1.2 m. A buffer layer and a rockfall catchment trench are arranged behind the barrier. The barrier has a front slope ratio of 1:0.10, while the buffer layer has a top width of 0.6 m and is filled with gravel soil along the slope surface. The buffer layer behind the barrier is backfilled with bagged crushed-stone soil or gravel soil containing pebbles and crushed stones, with a coarse-particle content greater than 30%. The slope ratio of the buffer layer is 1:0.75. Owing to long-term rainfall erosion, some rockfall barriers have lost their buffer layers. After rockfall impact, surface spalling occurred, and the exposed steel reinforcement became corroded, as shown in Figure 15c,d. In addition, the rainfall intensity was determined to evaluate the erosion resistance of the buffer material. Based on rainfall data provided by the local meteorological bureau, the rainfall intensity for the erosion-resistance test was set to 50 mm/h.

### 4.2. Erosion Resistance Characteristics

#### 4.2.1. Indoor Model Testing

To ensure that the deformation process in the model closely mimics real-world engineering behavior, the model was designed according to similarity theory, including geometric, physical, kinematic, and dynamic similarity. However, achieving complete similarity between the prototype and the model is challenging because of limitations in material properties, testing equipment, and experimental conditions. Therefore, a partial similitude approach was adopted in this study.

The primary objective of this study was to comparatively evaluate the rainfall-induced erosion resistance of ordinary gravelly soil and polyurethane–fiber modified gravelly soil rather than to achieve a strict quantitative simulation of the prototype. Accordingly, priority was given to preserving the key physical and mechanical properties governing erosion behavior, including density (*ρ*), and internal friction angle (φ). The shear strength parameters were primarily calibrated to ensure similarity of the internal friction angle, after which the cohesion value closest to the design target was selected as the final experimental parameter set. Therefore, the similarity constants were taken as *Cρ* = *C*φ=1. Meanwhile, to satisfy the experimental scale requirements while maintaining the geometric characteristics of the prototype, the geometric similarity constant was selected as ($l_{p}$/$l_{m}$ =2).

Here, *l*_p_ and *l*_m_ denote the characteristic lengths of the prototype and the model, respectively, while *γ*_p_ and *γ*_m_ denote the unit weights of the prototype and model materials, respectively. Thus, under the condition that the gravitational acceleration similarity ratio is unity (*C*g = 1), the unit weight similarity ratio is defined as $\gamma_{p}$/ = 1. The model was geometrically scaled according to the dimensions of the field buffer layer while maintaining the original slope ratio of 1:0.75. The internal friction angle (φ) is an angular parameter and was kept identical between the prototype and the model; therefore, its similarity constant was taken as 1. Since strain (ε) and Poisson’s ratio (μ) are dimensionless quantities, their similarity constants were also taken as 1. The similarity relationships and conversion factors for the remaining physical quantities are summarized in Table 8, while the derivation of the adopted similarity ratios is provided in Appendix A.

The variables in the equations are defined as follows: *l* denotes the model dimension, *γ* unit weight, *g* the gravitational acceleration, *c* the cohesion, *φ* the internal friction angle, *E* the model’s Young’s modulus, *δ* the displacement, *σ* the stress, *ε* the strain, *μ* Poisson’s ratio, *t* time, and *F* force. The similarity constants are: *C_σ_* for stress, *C_γ_* for density, *C_E_* for Young’s modulus, *C_ε_* for strain, *C_δ_* for displacement, *C_F_* for force, *C_c_* and for cohesion.

**Figure 16:**
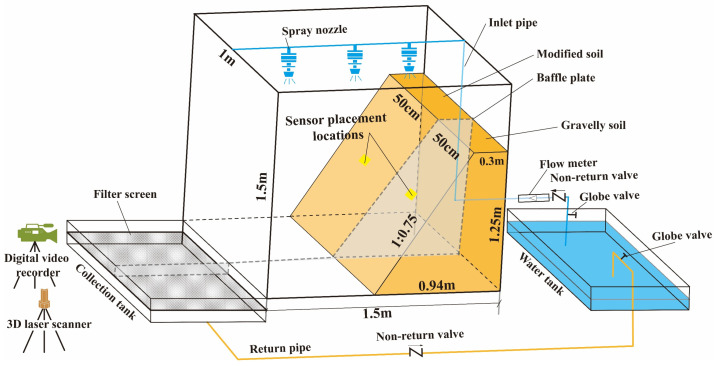
Schematic Representation of the Erosion Test Design.

To ensure both contrast and experimental rigor, ordinary gravel soil and modified gravel soil were separately placed in the model box to observe erosion degradation under rainfall-induced washout, as shown in Figure 16.

The experimental setup consists of a model box (1.5 m × 1 m × 1.5 m), nozzles, a water tank, a pump, pipes, and a flow meter. During the experiment, water is pumped from the tank to the nozzles, which release it to simulate rainfall. Rainfall intensity is controlled and stabilized via valve adjustments.

The measurement system consisted of moisture sensors, a video camera, a soil moisture data logger, and a 3D laser scanner. The moisture sensors were positioned at depths of 5 cm and 10 cm below the slope surface to monitor variations in moisture content within the buffer material. The video camera was used to record the rainfall erosion process, while the 3D laser scanner was used to characterize the surface deformation and morphological evolution of the buffer material after rainfall erosion.

Surface runoff does not form instantaneously, and its erosive impact on the soil is significant. Therefore, a sufficient rainfall duration is necessary to create the conditions for effective erosion. In the experiment, data were recorded every 10 min, with a total rainfall duration of 60 min. The rainfall intensity was set based on the worst-case scenario, using the maximum recorded local rainfall intensity. Based on meteorological data, the simulated rainfall intensity for the erosion experiment was set to 50 mm/h. It should be noted that the erosion test in this study was conducted under only one representative rainfall condition, with a rainfall intensity of 50 mm/h and a duration of 60 min. This condition was selected based on the local maximum historical rainfall record to simulate short-duration intense rainfall that may occur in mountainous areas and to provide a preliminary evaluation of the erosion resistance of the modified gravelly soil buffer layer. Under this condition, the modified soil maintained its overall structural integrity, with only slight detachment of surface particles. This result indicates that the polyurethane–fiber composite modification effectively improved the erosion resistance of the gravelly soil under the tested rainfall condition.

#### 4.2.2. Results of the Experiments

##### Characteristics of Erosion

**Figure 17:**
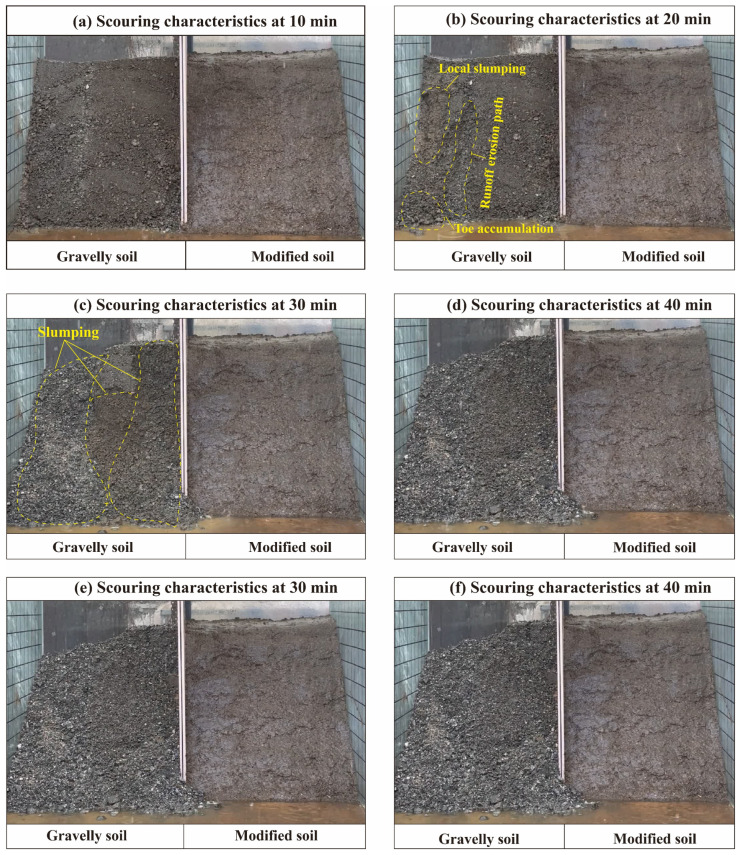
Evolution of rainfall-induced scouring and surface failure characteristics of gravelly soil and modified soil during the erosion test.

The erosion and failure characteristics of both gravel and modified gravel soils under varying rainfall durations are illustrated in Figure 17. The results demonstrate that after 10 min of rainfall, the surface of the ordinary gravel soil slope exhibited minor erosion, with the topsoil starting to peel away. In contrast, no significant erosion was observed in the modified gravel soil.

When the rainfall duration was 20 min, the infiltration rate of the rainwater was lower than the rainfall intensity, causing the water to gradually accumulate and form runoff. The erosive action of the surface runoff led to the formation of gullies on the surface of the gravel soil. Additionally, localized slope failures and gravel detachment were observed on the slope under the rain’s impact, with soil particles ultimately being transported to the base of the slope and forming an accumulation. In contrast, the modified gravel soil slope remained intact, showing no erosion damage, and no soil particle accumulation was observed at the foot of the slope.

After 30 min of rainfall, significant erosion occurred on the gravel soil slope. Washout gullies formed on the surface, and landslides were observed, leading to the displacement of soil particles which accumulated at the base of the slope. In contrast, the modified gravel soil slope remained largely intact, with no significant erosion or deformation.

After 40 min of rainfall, the gravel soil slope underwent severe damage, with the upper soil mass completely sliding down to the base of the slope. This resulted in significant deformation and continued particle loss. In contrast, the modified soil showed no noticeable damage, with only a small amount of surface particles or minor sections of consolidated material detaching due to the rainfall erosion, demonstrating excellent overall erosion resistance.

**Figure 18:**
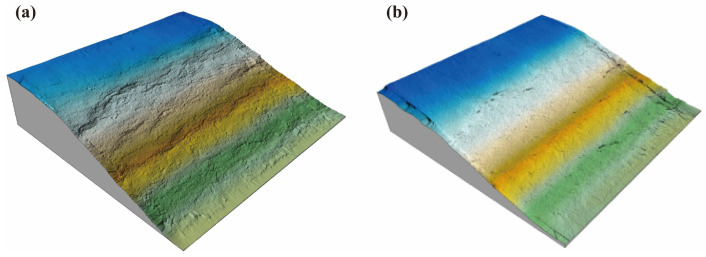
Comparative Analysis of 3D Scanning Features of Modified Soil Before and After the Experiment: (**a**) pre-experiment 3D scanning result; (**b**) post-experiment 3D scanning result. The color gradient represents elevation, where blue indicates lower elevations and yellow/green indicates higher elevations.

Additionally, to further quantify the morphological evolution of the modified soil slope under rainfall-induced erosion, a 3D laser scan was conducted on the slope surface following the rainfall experiment, as shown in Figure 18. A comparison of the pre- and post-experiment 3D terrain models reveals that the overall geometric profile and elevation distribution of the slope remained largely consistent. No significant gully formation, localized collapse, or large-scale material migration was observed. These results indicate that the modified soil demonstrates a high resistance to erosion and maintains stability under rainfall conditions.

Overall, the implemented improvements significantly enhanced the soil structure’s integrity and its resistance to erosion, enabling the slope surface to maintain both its intact macroscopic form and stable microscopic structure after rainfall-induced erosion.

##### Rainwater Infiltration, Runoff, and Soil Loss

**Figure 19:**
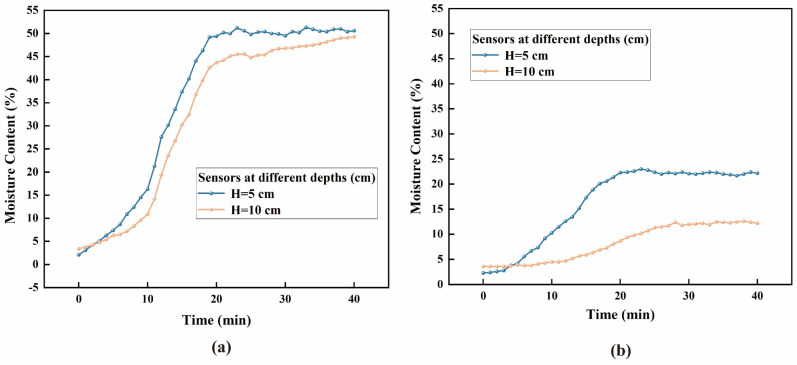
Variation in Moisture Content of Ordinary and Enhanced Gravel Soil: (**a**) Ordinary Gravel Soil; (**b**) Enhanced Gravel Soil.

Figure 19 shows the variation in moisture content of both ordinary gravel soil and modified gravel soil as a function of rainfall duration. The results reveal that ordinary gravel soil reaches near-saturation after 20 min of continuous rainfall, with a moisture content of approximately 50%. In contrast, for modified gravel soil, moisture content at depths of 5 cm and 10 cm after 20 min of rainfall was 20% and 11%, respectively, with subsequent changes stabilizing. This indicates that water infiltration is notably impeded, highlighting the strong impermeability of modified gravel soil to rainfall. Consequently, modified gravel soil enhances the erosion resistance of the buffer material, thereby maintaining the structural stability.

**Figure 20:**
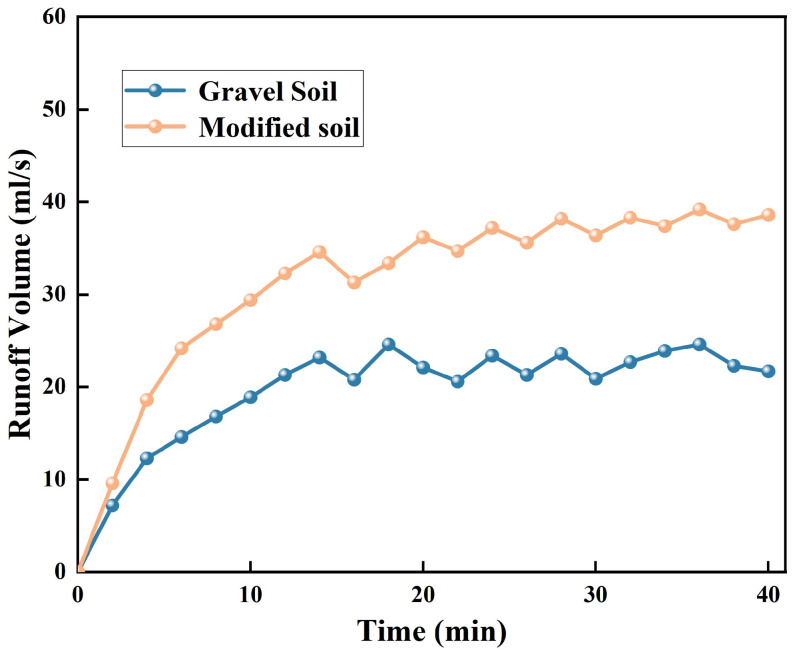
Patterns of Variation in Hillslope Runoff.

The development characteristics of slope surface runoff reflect the erosion resistance of the buffer material, and its temporal variation is shown in Figure 20. Initially, the soil is unsaturated, allowing rapid infiltration of rainwater. Since the infiltration rate exceeds the rainfall intensity, no runoff forms on the slope surface. As rainfall continues, the soil moisture content increases, and the infiltration rate decreases. Once the infiltration rate becomes lower than the rainfall intensity, runoff begins to develop on the slope surface.

Further analysis indicates that untreated gravel soil has relatively high porosity, allowing rainfall to infiltrate readily. As a result, slope runoff forms later and the runoff volume remains relatively low. With continued infiltration, the internal moisture content of the untreated gravel soil increases rapidly, reducing the effective stress and shear strength between particles. Under the combined effects of slope runoff and self-weight, the soil is therefore more prone to particle detachment, rill development, and local shallow collapse. In contrast, in the improved gravel soil, polyurethane fills the soil pores and forms a cemented structure, while the fiber-induced improvement in particle gradation also reduces soil porosity. These effects significantly reduce the rainfall infiltration rate and result in a relatively larger runoff volume. Although increased slope runoff may intensify surface hydrodynamic erosion, polyurethane cementation and fiber reinforcement substantially improve the detachment resistance of surface particles and enhance the overall stability of the soil, making the particles difficult to transport by runoff. Therefore, the improved gravel soil exhibits a behavior characterized by restricted infiltration, increased runoff, and substantially reduced particle loss. This indicates that the improved erosion resistance mainly results from enhanced material structural stability rather than simply from reduced slope runoff.

**Figure 21:**
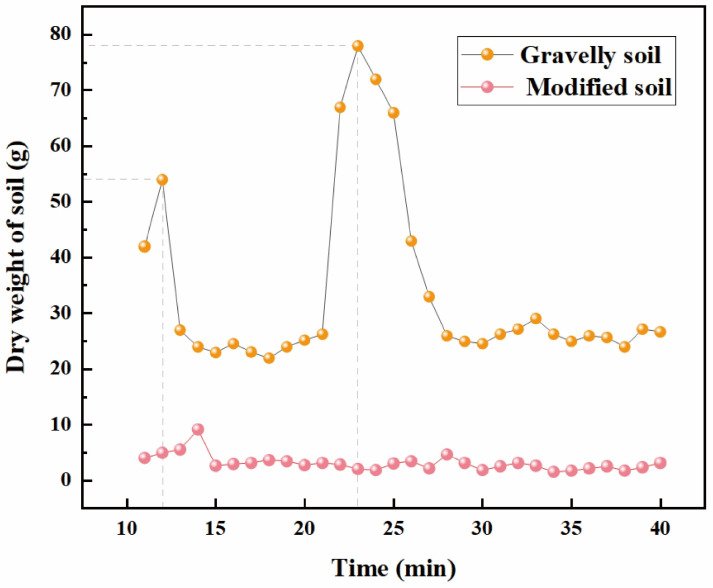
Dry Weight of Soil Lost per Minute for Different Soil Types During Erosion Process.

Figure 21 illustrates the variation in the dry weight of soil lost per minute during the rainfall erosion test for different soil types. The results show that ordinary gravel soil reaches distinct peak values at 12 min and 24 min, with dry soil loss of 54.1 g and 78.4 g, respectively. In contrast, the improved gravel soil experiences relatively low and stable soil loss throughout the test. These findings indicate that the stability of the gravel soil modified with polyurethane and fibers is significantly enhanced, effectively mitigating the erosive impact of rainfall runoff and reducing particle loss.

### 4.3. Buffering Capacity

Section 4.2 evaluates the erosion resistance of polyurethane-polypropylene fiber modified gravel soil through rainfall tests. The results demonstrate that it exhibits strong erosion resistance, effectively withstanding the erosive effects of rainwater. This section primarily focuses on evaluating its buffering performance.

#### 4.3.1. Model Test

Potential collapse materials are prone to detaching from the slope and falling under external forces such as rainfall, earthquakes, or human activities. During their movement, the collapsed rocks can bounce and roll upon contact with the slope, resulting in random rockfall behavior. Therefore, conducting model tests on the impact load of a rockfall barrier-buffer layer combination requires identifying and simplifying the characteristics of the rockfall impact load. Given the constraints of the model test conditions, the rockfall impact load was simplified in this experiment: the rockfall was modeled as a regular sphere with a defined mass, air resistance during its movement was neglected, and the direction and angle of impact were standardized to a horizontal direction.

**Figure 22:**
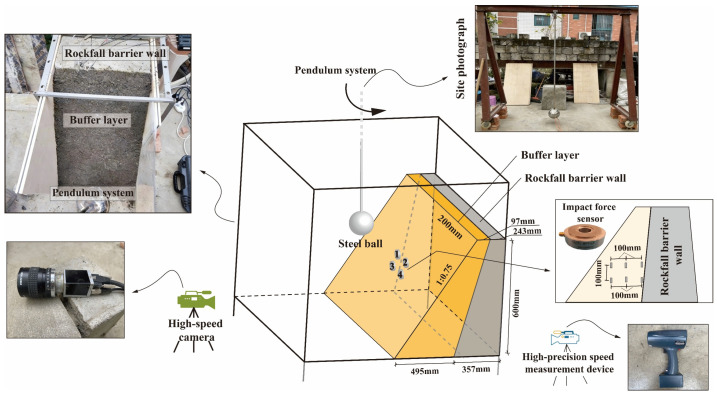
Schematic Representation of the Model Experiment. Arrows indicate the correspondence between labels and supplementary photographs (e.g., pendulum system, high-speed camera, high-precision speed measurement device). The yellow shaded area represents the gravel soil buffer layer. Numbers 1–4 indicate the locations of the four impact force sensors on the buffer layer surface.

The impact test is based on the rockfall barrier prototype used in the study area, as shown in Figure 15 To align the model test more closely with real-world conditions, a similarity ratio of 1:6 was applied, considering site limitations, as shown in Figure 22 The dimensions of the wall model are 357 mm × 243 mm × 600 mm (base × top × height). For model stability, the buffering layer and wall were simplified to the same height. The buffering layer model has dimensions of 495 mm × 97 mm × 600 mm (base × top × height). An external pendulum impact system was used for the impact tests (see Figure 22). The system consists primarily of a steel ball (approximately 200 mm in diameter, weighing 20 kg), a pendulum arm (hollow steel pipe, 1.8 m long), and a steel frame (2.4 m high, 2.2 m wide, welded from channel steel).

For data monitoring, three layers of impact force sensors are installed, spanning from the buffering layer to the rockfall barrier wall. The sensors are of model AFT-L2, with a measurement range of 50 kN and a natural frequency of 75 kHz. Data collection is performed using a 16-channel dynamic acquisition instrument, model AFT-2011. Each layer contains four measurement points. The first layer of sensors includes 1-1, 1-2, 1-3, and 1-4, totaling 12 impact force sensors (from 1-1 to 3-4). The horizontal, vertical, and interlayer spacings are all 100 mm. Additionally, a high-speed camera (model ACA2040-25GM) and a high-precision speedometer (model SVR400) are also installed.

#### 4.3.2. Impact Testing Plan

To explore the randomness of rockfall movement, the experiment simplifies the load characteristics and focuses on the cumulative impact effects of rocks with different energy levels at the same location on the buffering layer. The rockfall is modeled as a regular steel ball with a mass of 20 kg and a diameter of 200 mm, with air resistance and impact angle effects ignored. A pendulum system, shown in Figure 22, is used to apply horizontal orthogonal impact loads. Two material types—ordinary gravel soil and modified gravel soil—are tested under two conditions, with 10 impacts performed for each condition. In Table 8, Group I represents the ordinary gravelly soil buffer layer, whereas Group II represents the polyurethane–polypropylene fiber modified gravelly soil buffer layer. The impact location is fixed at 0.3H from the base of the wall. The pendulum release height is varied from 1.0H to 4.0H in 0.5H increments, creating a seven-level energy gradient. The initial impact velocity ranges from 2.8 m/s to 6.4 m/s, as detailed in the loading scheme in Table 9.

#### 4.3.3. Results and Analysis of the Experiment

##### Impact Dynamics and Velocity Damping Characteristics

**Figure 23:**
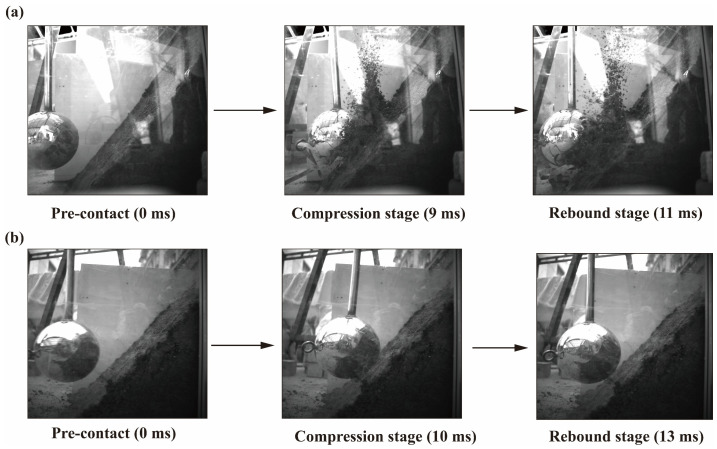
Impact Behavior of Different Buffer Layer Materials (arrows indicate temporal evolution): (**a**) Conventional Gravel Soil; (**b**) Enhanced Gravel Soil.

The typical impact process, recorded by high-speed cameras, reveals significant differences in the dynamic response of the two materials at the 4H energy level. The gravel soil buffering layer reaches its maximum compression depth 9 ms after impact and rebounds at 11 ms. In contrast, the modified gravel soil buffering layer experiences compression over a longer period, reaching maximum compression at 10 ms and rebounding at 13 ms, showing more pronounced ductile behavior. The impact process is shown in Figure 23.

The velocity change before and after the impact was measured using a speedometer, as presented in Table 10. The velocity restitution coefficient e is calculated using Equation (6).

(6) $$e=v_{2}/v_{1}$$

In this context, $v_{1}$ denotes the velocity of the steel ball as it approaches the buffering material, $v_{2}$ represents the velocity of the steel ball after leaving the buffering material, and e is the velocity restitution coefficient.

The velocity change before and after impact shows that the velocity attenuation rate for gravel soil ranges from 45.9% to 57.1%, while for the modified soil, the attenuation rate significantly increases to between 65.6% and 71.4%, indicating superior buffering performance. As the rockfall height and impact energy increase, the velocity attenuation rates of both materials exhibit a nonlinear decrease, reflecting the saturation effect of energy dissipation in the buffering material under high-energy impacts. The improved velocity reduction capability of the modified soil is due to the elastic energy dissipation of the polyurethane polymer and the stress distribution effect of the fiber framework. Additionally, the polyurethane’s inherent viscoelasticity effectively prolongs the energy dissipation time and allows it to absorb some of the impact energy through plastic deformation.

##### Impact Force

**Figure 24:**
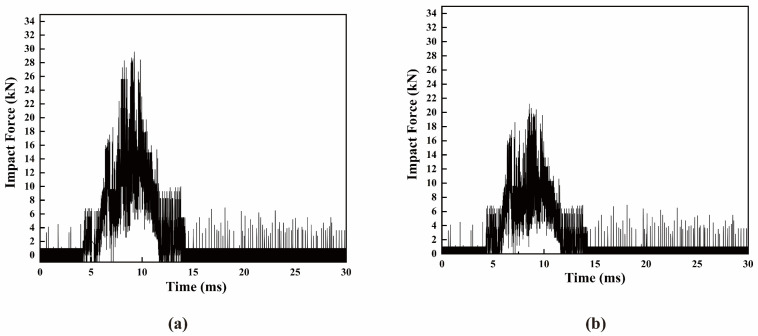
Time-History Curves of Peak Impact Force for Different Buffer Layer Materials: (**a**) Conventional Gravel Soil; (**b**) Enhanced Gravel Soil.

The rockfall impact forces under different conditions were calculated based on measurements from the embedded impact force sensors within the buffering material. The maximum impact force was selected for analysis, and the time-history curves of the peak impact forces for different buffering materials are shown in Figure 24.

The impact force time-history curve clearly exhibits nonlinear characteristics, which can be broadly divided into three phases: a linear growth phase, a nonlinear peak phase, and a stabilization phase after unloading. The results indicate that the maximum peak impact force on the gravel soil buffering layer is 30.8 kN, while the maximum peak impact force on the modified soil buffering layer is reduced to 21.2 kN, showing a significant decrease. This reduction is attributed to the favorable hydrogel properties of the polyurethane polymer, which fills the gaps between particles, acting like a “cushion” and effectively diminishing the transmission of impact stress.

**Figure 25:**
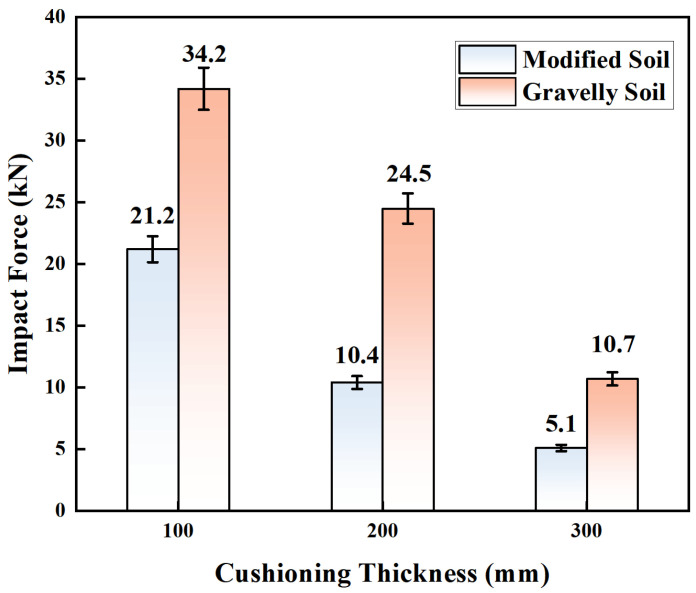
Variation in Peak Impact Force with Changes in Buffer Layer Thickness.

To investigate the impact of buffering material thickness on the impact force, three layers of impact force sensors were placed, labeled 1-1 to 1-4, 2-1 to 2-4, and 3-1 to 3-4, with a 100 mm spacing between layers. The distance from the impact surface to sensors 1-1 and 1-2 was also 100 mm. The arrangement of sensors is shown in Figure 22. The relationship between the maximum impact force and the buffering layer thickness is presented in Figure 25. The results indicate that increasing the thickness of the buffering material effectively reduces the peak impact force on the structure. The impact force reduction in the modified soil buffering layer is relatively uniform, whereas for the gravel soil buffering layer, the reduction becomes more pronounced when the thickness exceeds 200 mm. This further highlights that increasing the buffering layer thickness is an effective strategy for improving energy absorption performance.

##### Analysis of the Deformation Characteristics of the Buffer Layer

**Figure 26:**
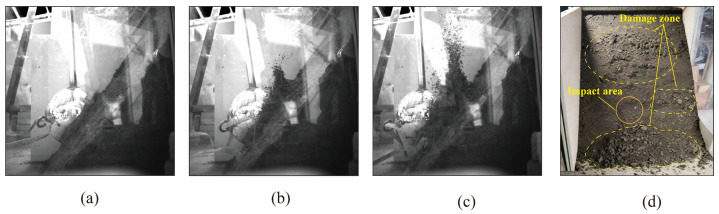
Deformation and Failure Modes of the Gravel Soil Buffer Layer: (**a**) Compaction Due to Impact; (**b**) Crack Formation; (**c**) Impact-Induced Damage; (**d**) Post-Impact Buffer Layer Slope Deformation.

Real-time capture of the entire rockfall impact process revealed that the deformation of the gravel soil buffering layer exhibited clear staged characteristics: compaction, crack development, and failure stages, as shown in Figure 26. Upon impact, local volumetric compression occurred at the load center, accompanied by the ejection of a small number of surface particles, with the structure maintaining its overall form. As the rockfall penetration depth increased, stress around the impact point exceeded the frictional resistance between particles, resulting in the formation of through cracks in the slope. Local sliding occurred along the crack surface, and the range of particle ejection rapidly expanded. Ultimately, the buffering layer experienced large-scale sliding crack formation and gravel peeling, resulting in overall brittle failure. In contrast, the solidified soil material exhibited no significant damage during the impact process, maintaining its overall integrity.

**Figure 27:**
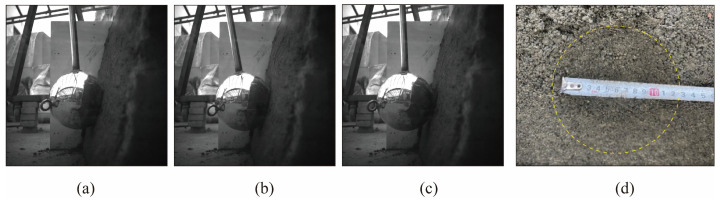
Deformation and Failure Modes of the Gravel Soil Buffer Layer: (**a**) Impact Moment; (**b**) Compaction Under Impact; (**c**) Pendulum Rebound; (**d**) Post-Impact Indentation (yellow circles indicate the boundary of the deformed area).

The modified gravel soil material exhibited no significant damage during the impact process and maintained its overall integrity, as shown in Figure 27 Observations during the experiment revealed that no noticeable crack expansion occurred on the slope surface. Instead, local compressive deformation appeared near the impact point, with a plastic pit approximately 10 cm in diameter and 2 cm in depth. Apart from a small number of incompletely consolidated particles detaching, the overall structure remained intact throughout the process.

In conclusion, the polyurethane and polypropylene fiber composite-modified gravel soil exhibits excellent impact resistance as a buffer layer for rockfall barriers, with a notable improvement over conventional gravel soil buffer layers.

## 5. Discussion and Conclusions

### 5.1. Discussion

Recycled polypropylene fibers enhance sustainability by valorizing waste polymers and reducing reliance on virgin fibers, aligning with circular geotechnical material development [36].

This study primarily focuses on the stabilization effects of recycled polypropylene fibers and polyurethane on gravelly soil. Nevertheless, the preparation of recycled polypropylene fibers warrants further discussion. At present, recycled polypropylene fibers are mainly prepared through two routes: mechanical recycling of waste plastic products and reprocessing of plastic waste by extrusion or melt processing to produce fibers with more controllable dimensions and surface morphologies [44]. Mechanically cut fibers generally retain the surface and dimensional characteristics of the original products, which may result in considerable variability in fiber diameter, aspect ratio, surface roughness, and tensile properties [45]. During melt reprocessing, chain scission and oxidative reactions can reduce molecular weight, decrease melt viscosity, alter viscoelastic behavior, and ultimately affect fiber-forming stability and mechanical quality [46]. Therefore, the key issue in recycled PP fiber preparation is not only how to reuse polypropylene waste, but also how to maintain melt stability and fiber quality during repeated processing.

To address this issue, increasing attention has been directed toward stabilization strategies during the melt processing of recycled polypropylene. In addition to conventional synthetic stabilizers, natural antioxidants have been considered a more environmentally friendly alternative. Shambilova et al. introduced Vitamin E as a natural antioxidant into polypropylene melts and demonstrated that antioxidant-assisted processing can reduce thermomechanical and oxidative degradation during repeated processing [46]. This antioxidant-assisted route provides a more environmentally benign option for conventional melt-processing methods. Future preparation of recycled polypropylene fibers should move toward cleaner and more controllable processing routes, including waste sorting, mechanical recycling, melt stabilization, and fiber-forming optimization. Among these strategies, natural-antioxidant-assisted melt processing represents a promising direction for producing environmentally friendly recycled polypropylene fibers.

From the perspective of environmentally friendly composite material design, cellulose fibers and silica nanoparticles also show potential [47]. Cellulose fibers are renewable and low-carbon, making them sustainable alternatives to synthetic fibers. However, their hydrophilicity, dimensional variability, and potential degradation under wetting–drying cycles or alkaline environments may introduce uncertainty into long-term performance [48,49]. Silica nanoparticles may improve matrix densification and interfacial characteristics owing to their high specific surface area and filling effect, but their effectiveness strongly depends on dispersion quality and dosage control [50,51]. Nanoparticle agglomeration may reduce material uniformity and increase processing complexity. Therefore, cellulose fibers and silica nanoparticles were not included in the experimental design of this study. Future research may further explore surface-treated cellulose fibers, nano-silica-modified polymer systems, or natural-antioxidant-stabilized recycled polypropylene fibers to develop more environmentally friendly and compatible composite systems.

Although the ESEM observations indicate that excessive polyurethane may form relatively thick coating layers around particle aggregates, the present study does not provide direct quantitative evidence linking this microstructural feature to the observed reduction in mechanical performance. Therefore, the proposed mechanism should be regarded as a possible interpretation based on qualitative observations rather than a definitive conclusion. Future studies involving interparticle friction measurements, interface characterization, and quantitative image analysis are required to clarify the influence of excessive polyurethane on particle interaction and load-transfer behavior.

It should be acknowledged that the erosion resistance evaluation in this study was conducted under a single rainfall intensity and duration condition. While this scenario represents a severe short-term rainfall event in mountainous regions, it does not capture the full variability of natural precipitation. Therefore, the current results mainly reflect the performance under a representative extreme condition rather than general rainfall behavior. Future work should consider multiple rainfall intensities, durations, and cyclic rainfall conditions to comprehensively assess the long-term erosion resistance of the improved gravel soil.

The present study demonstrates the effectiveness of polyurethane–fiber stabilization for gravel soil improvement, no direct comparison with conventional stabilization methods (e.g., cement, lime, geopolymers, basalt fibers, or virgin polypropylene fibers) was conducted under identical testing conditions. Therefore, the relative performance advantage of the proposed method should be interpreted within the scope of this study. Future research will focus on systematic benchmarking against commonly used stabilization techniques to further evaluate its engineering competitiveness and applicability.

### 5.2. Conclusions

This study systematically investigated the mechanical behavior and reinforcement mechanisms of gravelly soil stabilized with waterborne polyurethane and recycled polypropylene fibers. The main conclusions are summarized as follows:

(1) Polyurethane and recycled polypropylene fibers substantially improved the mechanical properties of gravelly soil. At the optimal mixture proportion of 6.8% polyurethane and 0.19% fiber, the unconfined compressive strength increased from 107.6 kPa to 931.5 kPa, corresponding to an increase of 765.7%. The cohesion increased from 23.4 kPa to 83.44 kPa, representing an increase of 256.4%, while the internal friction angle increased from 43.4° to 61.23°, corresponding to an increase of 41.08%.
(2) Response surface analysis showed that polyurethane and fiber contents had pronounced nonlinear effects on all mechanical indices. These effects were mainly associated with the bonding and pore-filling effects of polyurethane and the reinforcing effect of fibers. Within the experimental range considered in this study, the interaction between polyurethane and fiber content was not significant.
(3) Polyurethane enhanced interparticle bonding by forming cementing films on particle surfaces and filling pore spaces. The fibers improved the structural integrity and deformation resistance of gravelly soil through interparticle friction, mechanical interlocking, and bridging effects, thereby enhancing its mechanical performance.
(4) The modified gravelly soil exhibited stronger erosion resistance under rainfall erosion and showed improved energy dissipation capacity and more stable velocity attenuation under impact loading. These results indicate that the modified gravelly soil has good engineering applicability as a buffer layer material.
(5) Although the effectiveness of the proposed material was verified under laboratory conditions, this study still has limitations, particularly the insufficient consideration of long-term service environments. Future research should focus on recycled polypropylene fibers with complex geometries, such as crimped or split fibers, and surface-functionalized modifications. These approaches may substantially improve fiber dispersion uniformity and interfacial bonding efficiency, thereby further enhancing the overall engineering performance and reliability of the composite stabilization system.

## Figures and Tables

**Table 1:** Basic physicochemical properties of the waterborne polyurethane.

Polyurethane Type	Appearance	Solid Content (%)	Main Component	Density (g/cm^3^)	NCO (%)	Viscosity (mPa·s)
Waterborne polyurethane	Colorless transparent liquid	99.9	NCO-containing polyurethane prepolymer	1.0	22.5	2000–6000

Note: the basic physical properties of the materials were provided by the supplier. NCO (isocyanate group): a reactive functional group (–N=C=O) in polyurethane prepolymers that determines curing and crosslinking ability.

**Table 2:** Mechanical properties of recycled polypropylene fibers and virgin polypropylene fibers.

Fiber Type	Tensile Strength (MPa)	Elongation at Break (%)	Elastic Modulus (GPa)	Density (g/cm^3^)	Nominal Average Diameter (µm)	Melting Point (°C)
Recycled polypropylene fiber	325	60.6	3.3	0.91	33	169
virgin polypropylene fiber	469	74.4	3.5	0.90	33	170

Note: the basic physical properties of the materials were provided by the supplier (Hebei Taihui Plastic Products Co., Ltd., Shijiazhuang, China).

**Table 3:** Mixture design and specimen identification.

Specimen ID	Polyurethane (PU, wt.%)	Fiber (F, wt.%)
PU0-F0	0	0
PU2-F0	2	0
PU2-F0.1	2	0.1
PU2-F0.2	2	0.2
PU2-F0.3	2	0.3
PU4-F0	4	0
PU4-F0.1	4	0.1
PU4-F0.2	4	0.2
PU4-F0.3	4	0.3
PU6-F0	6	0
PU6-F0.1	6	0.1
PU6-F0.2	6	0.2
PU6-F0.3	6	0.3
PU8-F0	8	0
PU8-F0.1	8	0.1
PU8-F0.2	8	0.2
PU8-F0.3	8	0.3
PU10-F0	10	0
PU10-F0.1	10	0.1
PU10-F0.2	10	0.2
PU10-F0.3	10	0.3

Note: the symbol “%” represents mass fraction (wt.%).

**Table 4:** Central composite design experimental results.

No.	F (%)	PU (%)	c (kPa)	φ (°)	Unconfined Compressive Strength (UCS) (kPa)
1	0.0	0	23.4	43.4	106.4
2	0.3	0	34.1	46.3	134.1
3	0.0	10	46.0	45.1	621.2
4	0.3	10	72.0	52.4	732.8
5	0.0	6	29.63	46.9	697.5
6	0.3	6	76.4	57.4	832.5
7	0.2	0	68.41	50.1	462.4
8	0.2	10	69.0	56.3	803.1
9	0.2	6	86.1	61.2	960.3
10	0.2	6	80.7	59.5	901.2
11	0.2	6	82.5	60.9	948.0
12	0.2	6	77.6	58.8	912.1
13	0.2	6	84.8	60.5	975.0

Note: F denotes recycled polypropylene fiber content; PU denotes polyurethane content; c denotes cohesion; φ denotes internal friction angle; and UCS denotes unconfined compressive strength.

**Table 5:** Coefficient significance and analysis of variance for the unconfined compressive strength regression model.

Source of Variation	F-Value	*p*-Value	Coefficient of Retained Term
Model	58.21	<0.0001	132.69
*x*_1_ (Fiber)	27.82	0.0012	3051.45
*x*_2_ (PU)	100.31	<0.0001	165.78
*x*_1_^2^	26.19	0.0014	−9391.52
*x*_2_^2^	63.05	<0.0001	−11.95
*x*_1_ *x*_2_	0.12	0.742	-
*R*^2^ = 0.98, Adjusted *R*^2^ = 0.96			

**Table 6:** Coefficient significance and analysis of variance for the cohesion regression model.

Source of Variation	F-Value	*p*-Value	Coefficient of Retained Term
Model	11.77	0.0027	22.60
*x*_1_ (Fiber)	19.23	0.003	408.47
*x*_2_ (PU)	3.92	0.088	5.28
*x*_1_^2^	14.48	0.007	−1124.04
*x*_2_^2^	2.61	0.150	−0.39
*x*_1_ *x*_2_	0.42	0.538	-
*R*^2^ = 0.90, Adjusted *R*^2^ = 0.82			

**Table 7:** Coefficient significance and analysis of variance for the internal friction angle regression model.

Source of Variation	F-Value	*p*-Value	Coefficient of Retained Term
Model	49.99	<0.0001	42.35
*x*_1_ (Fiber)	55.98	<0.0001	104.50
*x*_2_ (PU)	40.53	<0.0001	2.54
*x*_1_^2^	47.04	<0.0001	5.54
*x*_2_^2^	42.85	<0.0001	−0.24
*x*_1_ *x*_2_	3.76	0.094	-
*R*^2^ = 0.97, Adjusted *R*^2^ = 0.95			

**Table 8:** Similarity parameters between model and prototype.

Physical Quantity	Similarity Relation	Similarity Ratio	Remarks
Geometric length (*L*)	*l_p_* = $C_{l}$*L_m_*	2	Controlled quantity
Internal friction (φ)	φ*_p_* = φ*_m_*	1	Angular parameter
Cohesion (*c*)	*c_p_* = *C_σ_c_m_*	2	Same dimension as *σ*
Water content (*ω*)	*ω_p_* = *ω_m_*	1	Dimensionless
Unit weight (*γ*)	*γ_p_* = *C_γ_γ_m_*	1	Controlled quantity
Strain (*ε*)	*ε_p_* = *ε_m_*	1	Dimensionless
Elastic modulus (*E*)	*E_P_* = *C_σ_*/*C_E_E_m_*	2	*C_E_* = *C_σ_*
Poisson’s ratio (*μ*)	*μ_p_* = *μ_m_*	1	Dimensionless
Displacement (*δ*)	*δp* = *C_l_δ_m_*	2	Geometric similarity
Stress (*σ*)	*σ_p_* = *C_γ_C_L_σ_m_*	2	*C_σ_* = *C_γ_C_l_*
Force (*F*)	*F_p_* = *C_σ_C_l_*^2^*F_m_*	2^3^	*C_E_* = *C_l_*^3^
Flexural rigidity (*EI*)	*EI_p_* = *C_E_C_l_*^2^*(EI)_m_*	2^5^	*C_EI_* = *C_l_*^5^
Time (*t*)	*t_p_* = *C_l_*^1/2^*t_m_*	2^1/2^	Dynamic similarity
Gravitational Acceleration	g_p_ = *g*_m_	1	Same gravity field (no scaling)

**Table 9:** Loading Scheme.

Operating Condition Number		Rockfall Drop Height (cm)	Pre-Impact Velocity (m/s)
I-1	II-1	60 (1.0H)	2.8
I-2	II-2	90 (1.5H)	3.7
I-3	II-3	120 (2.0H)	4.4
I-4	II-4	150 (2.5H)	5.2
I-5	II-5	180 (3.0H)	5.6
I-6	II-6	210 (3.5H)	6.1
I-7	II-7	240 (4.0H)	6.4

Note: Group I denotes the ordinary gravelly soil buffer layer, and Group II denotes the polyurethane–polypropylene fiber modified gravelly soil buffer layer.

**Table 10:** Comparison of Impact Velocities Before and After Striking Gravelly Soil and Modified Gravelly Soil Cushion Layers.

**Comparison of Pre- and Post-Impact Velocity for Rockfall Buffer Material**				
Operating Conditions	$v_{1}$ (m/s)	$v_{2}$ (m/s)	Velocity Decay Rate (%)	e
I-1	2.8	1.2	57.1	0.43
I-2	3.7	1.6	56.7	0.43
I-3	4.4	2.2	50.0	0.50
I-4	5.2	2.4	53.8	0.46
I-5	5.6	2.9	48.2	0.52
I-6	6.1	3.3	45.9	0.54
I-7	6.4	3.4	46.9	0.53
**Comparison of Pre- and Post-Impact Velocity for Improved Rockfall Buffer Materials**				
II-1	2.8	0.8	71.4	0.29
II-2	3.7	1.1	70.3	0.30
II-3	4.4	1.3	70.5	0.30
II-4	5.2	1.6	69.2	0.30
II-5	5.6	1.8	67.9	0.32
II-6	6.1	2.1	65.7	0.34
II-7	6.4	2.2	65.6	0.34

## Data Availability

The original contributions presented in this study are included in the article. Further inquiries can be directed to the corresponding author.

## Abbreviations

The following abbreviations are used in this manuscript:

ESEM	Environmental scanning electron microscopy
CCD	Central composite design

## Appendix A. Derivation of Similarity Ratios

The development of similar materials is a prerequisite for physical model testing and is critical for ensuring that the model accurately represents the physical properties of the prototype. Based on similarity theory, quantitative relationships between the model and the prototype were established to describe the governing relationships of the physical system. To ensure that the deformation behavior of the model is consistent with that of the prototype, the model should satisfy the similarity criteria. The relevant physical parameters can be expressed by the following relationship, as shown in Equation (A1).

(A1) $$f=\left(l,\gamma ,g,c,\varphi ,E,\delta ,\sigma ,\varepsilon ,\mu ,t,F\right)=0$$

In Equation (A1), *l* is the characteristic length, γ is the unit weight, g is the gravitational acceleration, c is the cohesion, φ is the internal friction angle, E is the elastic modulus, δ is the displacement, σ is the stress, ε is the strain, μ is Poisson’s ratio, t is the time, and F is the force. Since φ, ε, and μ are dimensionless quantities, their similarity ratios are equal to 1; namely, $C_{\varphi }=C_{\mu }=C_{\varepsilon }=1$. By setting $C_{\gamma }=1$, the similarity relationships for the other physical quantities can be derived according to the criteria expressed in Equations (A2)–(A4).

(A2) $$C_{l}=l_{p}/l_{m}$$

where *C_l_* is the geometric similarity ratio, l denotes the characteristic length, and the subscripts p and m denote the prototype and the model, respectively.

(A3) $$\left\{\begin{array}{l} C_{\sigma }=C_{l}C_{\gamma }\\ C_{\sigma }=C_{E}C_{\varepsilon }\\ C_{\delta }=C_{l}\\ C_{F}=C_{\sigma }C_{l}^{2} \end{array}\right.$$

where *C_σ_*, *C_γ_*, *C_E_*, *C_ε_*, *C_δ_* and *C_F_* are the similarity ratios for stress, unit weight, elastic modulus, strain, displacement, and force, respectively.

(A4) $$\left\{\begin{array}{l} C_{\sigma }=C_{R}^{\tau }\\ C_{c}=C_{R}^{\tau }\\ C_{R}^{c}=C_{R}^{\tau } \end{array}\right.$$

where $C_{R}^{\tau }$, $C_{c}$, and $C_{R}^{c}$ are the similarity ratios for tensile strength, cohesion, and compressive strength, respectively.
